# Anti-Tumor Effects of the Peptide TMTP1-GG-_D_(KLAKLAK)^2^ on Highly Metastatic Cancers

**DOI:** 10.1371/journal.pone.0042685

**Published:** 2012-09-11

**Authors:** Xiangyi Ma, Ling Xi, Danfeng Luo, Ronghua Liu, Shu Li, Yan Liu, Liangsheng Fan, Shuangmei Ye, Wanhua Yang, Shuhong Yang, Li Meng, Jianfeng Zhou, Shixuan Wang, Ding Ma

**Affiliations:** Cancer Biology Research Center, Tongji Hospital, Tongji Medical College, Huazhong University of Science and Technology, Wuhan, Hubei, P. R. China; Mie University Graduate School of Medicine, United States of America

## Abstract

The treatment of cancer such as oligonucleotides or peptides requires efficient delivery systems. A novel peptide, TMTP1, previously derived and identified in our laboratory showed remarkable ability to target highly metastatic tumors both *in vitro and in vivo*, even at the early stage of occult metastasis foci. TMTP1 moderately inhibited tumor cell viability, although not enough to deem it an efficient killer of tumor cells. In this study, we sought to enhance the anti-tumor activity of TMTP1. To do this, we fused it to an antimicrobial peptide, _D_(KLAKLAK)^2^, and termed the resulting peptide TMTP1-DKK. We found that TMTP1-DKK could trigger rapid apoptosis in human prostate and gastric cancer cells through both the mitochondrial-induced apoptosis pathway and the death receptor pathway. Furthermore, direct injection of TMTP1-DKK into mice with prostate and gastric xenograft cancers resulted in reduction of tumor volumes and a significant delay in tumor progression and metastasis *in vivo*. These results suggest that TMTP1-DKK may serve as a powerful therapeutic agent for metastatic tumors.

## Introduction

The majority of mortality associated with cancer is due to metastasis of the original tumor cells to sites distant from the initial or primary tumor. Currently available treatment options are rarely able to cure metastatic cancers, such as those arised from prostate and gastric cancer. Worldwide, prostate cancer is the most common malignancy in men and the second leading cause of cancer-related deaths. Gastric cancer remains one of the most frequent neoplasms, causing 12% of all cancer-related deaths each year [1], [2], [3]. Furthermore, clinical treatment of these solid tumors remains relatively ineffective. Therefore, detecting and treating tumor metastasis, especially occult metastases, has been the main approach of newly developed cancer therapies. The past several years of cancer research has provided insights into the processes responsible for cancer growth and identified numerous molecular targets for possible cancer therapy [4], [5]. However, the approval procedure for new cancer therapies is lengthy. The hope is that by targeting specific alterations in cancer cells at the early stage of metastasis, therapies can be developed to be more effective in killing tumor cells *in situ* and metastasis foci, while less harmful to normal cells. As a result, these innovative therapies would make a major positive impact on the survival and quality of life of cancer patients.

Over the past decade, the field of cancer drug development has been transformed with the identification of specific molecular targets [6], [7], [8]. Targeted gene therapy can be achieved through a variety of techniques including gene therapy and gene transcription. Recent advantages in targeted delivery include the successful use of small molecular inhibitors, monoclonal antibodies, and short targeting peptides [9], [10]. The development of short targeting peptides seems to be a promising avenue for successful targeted gene therapy. Short targeting peptides have excellent tissue penetrability and minimal toxicity and immunogenicity, making them apt for acceptance by patients and clinicians.

Recently, we identified a 5-amino acid peptide, TMTP1, which bound to a series of highly metastatic cancer cell lines *in vitro* and *in vivo*, particularly those from atypical liver micrometasteses that contained small numbers of neoplastic cells [11]. However, TMTP1 did not recognize normal and non-metastatic cell lines. We also found that high concentrations of TMTP1 could mediate tumor cell apoptosis. Since TMTP1 bound to metastatic cancers with high specificity, it could be useful as a diagnostic tool and/or have cytotoxic function. Specifically, TMTP1 could be used in the construction of customizable *de novo* peptide conjugates. These peptides may be customized for various diagnostic and therapeutic applications through conjunction to a wide range of targeting agents such as viruses, proteins, and antimicrobial peptides. In this study, we coupled TMTP1 to a cationic antimicrobial peptide known for its strong cytotoxic activity in order to enhance its anti-tumor effects.

There are more than 100 naturally occurring antibiotic peptides and their *de novo* design has received much attention [12], [13], [14], [15], [16]. Ellerby et* al* [17] found that when they conjugated a cationic antimicrobial peptide, _D_(KLAKLAK)^2^, to the CNGRC homing domain, the resulting peptide had anti-tumor activity through its ability to target mitochondria and trigger apoptosis. These and other structurally similar pro-apoptotic antibiotic peptides remain relatively non-toxic outside of eukaryotic cells. However, once taken up by the tumor cells, they induced mitochondrial swelling and mitochondria dependent apoptosis [18], [19], [20]. However, a major problem with these peptides is ensuring their introduction into cells. Thus, these peptides must be coupled to tumor targeting peptides that allow receptor-mediated internalization. This ensures that the chimeric peptide will enter the cytosol of targeted cells where it may induce mitochondrial-dependent apoptosis.

In this study, we fused _D_(KLAKLAK)^2^ to TMTP1 by solid phase peptide synthesis on an automated Applied Biosystems model and named the resultant peptide TMTP1-DKK [11]. We hypothesized that the TMTP1 peptide would allow for targeting while _D_(KLAKLAK)^2^ would promote mitochondria induced apoptosis. In this study, we assessed the remarkable specificity and anti-tumor ability of TMTP1-DKK on metastatic tumors *in vitro* and *in vivo*.

## Results

### TMTP1-DKK homes to highly metastatic tumor cells in vitro and in vivo

**Figure 1:**
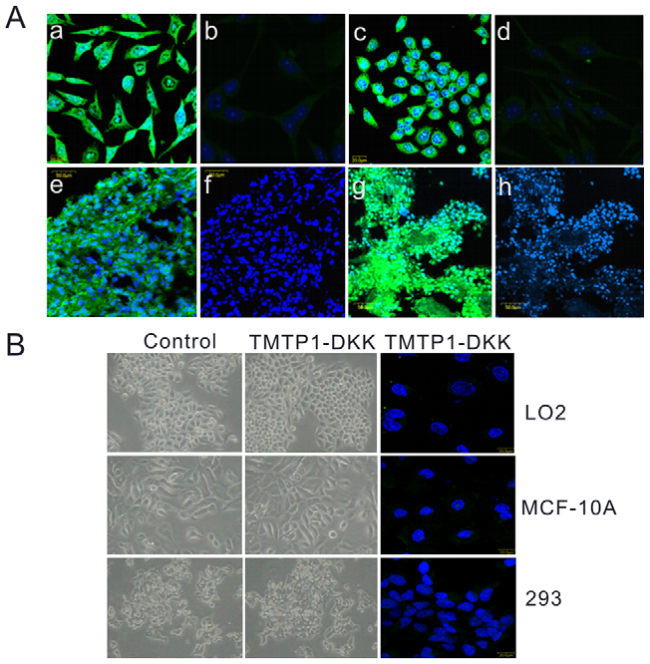
Specific targeting of TMTP1-DKK peptides *in vitro* and *in vivo*. A Fluorescent images of tumor cells treated with TMTP1-DKK were examined with Confocal laser scanning microscopy. (a) PC-3M-1E8 cells (b) PC-3M-2B4 cells (c) MKN-45sci cells (d) NIH/3T3 cells. FITC-TMTP1-DKK (green) and control peptide were examined in xenograft tumors including PC-3M-1E8 (e)TMTP1-DKK, (f)svTMTP1-DKK, MKN-45sci (g) TMTP1-DKK, (h) svTMTP1-DKK. Nuclei were co-stained with DAPI (blue). B Fluorescent images of nomal cells (normal mammary epithelial cell MCF-10A, normal liver cell LO2 and HEK293 cells) treated with TMTP1-DKK were examined with Confocal laser scanning microscopy. The morphological change of nomal cells was visualized using inverted microscope.

We have previously shown that TMTP1 binds specifically to metastatic tumor cells [11]. To determine if the newly synthesized TMTP1-DKK peptide retains the binding specificity of its targeting domain, TMTP1, we conjugated TMTP1-DKK to FITC. We then examined the binding of FITC-conjugated TMTP1-DKK to several cancer cell lines and normal cells *in vitro* and *in vivo*. FITC-conjugated TMTP1-DKK specifically bound to the highly metastatic prostate cancer cell line PC-3M-1E8 and the gastric cancer cell line MKN-45sci (Figure 1A, a and c,). However, TMTP1-DKK did not target the non-metastatic cell line PC-3M-2B4 cells, the murine fibroblast cell line NIH/3T3, normal mammary epithelial cell MCF-10A, normal liver cell LO2 and HEK293 cells (Figure 1A, b, d and B).

We next sought to determine whether TMTP1-DKK bound to these highly metastatic tumor cells *in vivo*. For this purpose, PC-3M-1E8 and MKN-45sci cells were subcutaneously injected into BALB/c nude mice. Mice with tumors 1–1.5 cm in diameter were injected with 300 µg FITC-TMTP1-DKK peptide via the tail vein. After 24 hours, tumors and the control organs were excised and processed for frozen sectioning. We detected the Dynamic biodistribution of FITC conjugated TMTP1-DKK from at 1 h, 6 h, 12 h, 24 h, and 48 h in the mouse models of MKN-45sci orthotopic gastric cancer and PC-3M-1E8 subcutaneous prostate cancer by confocal microscopy. TMTP1-DKK was detected in highly metastatic PC-3M-1E8 and MKN-45sci xenograft tumor tissues (Figure 1A, e and g). By confocal microscopy showed that TMTP1-DKK could persist at a higher level for 48 h in the mouse models of MKN-45sci orthotopic gastric cancer and PC-3M-1E8 subcutaneous prostate cancer **(Figure S1).** Which might indicated that the peptide could stable in tumor tissue. However, fluorescence was not detected in control organs and tissues. The homing assay with the control peptide, svTMTP1-DKK, showed no obvious fluorescence staining in PC-3M-1E8 and MKN-45sci xenograft tumor tissue or normal tissues (Figure 1A, f and h, **Figure S2,)**.

### TMTP1-DKK inhibits proliferation and induces apoptosis in highly metastatic cancer cells

**Figure 2:**
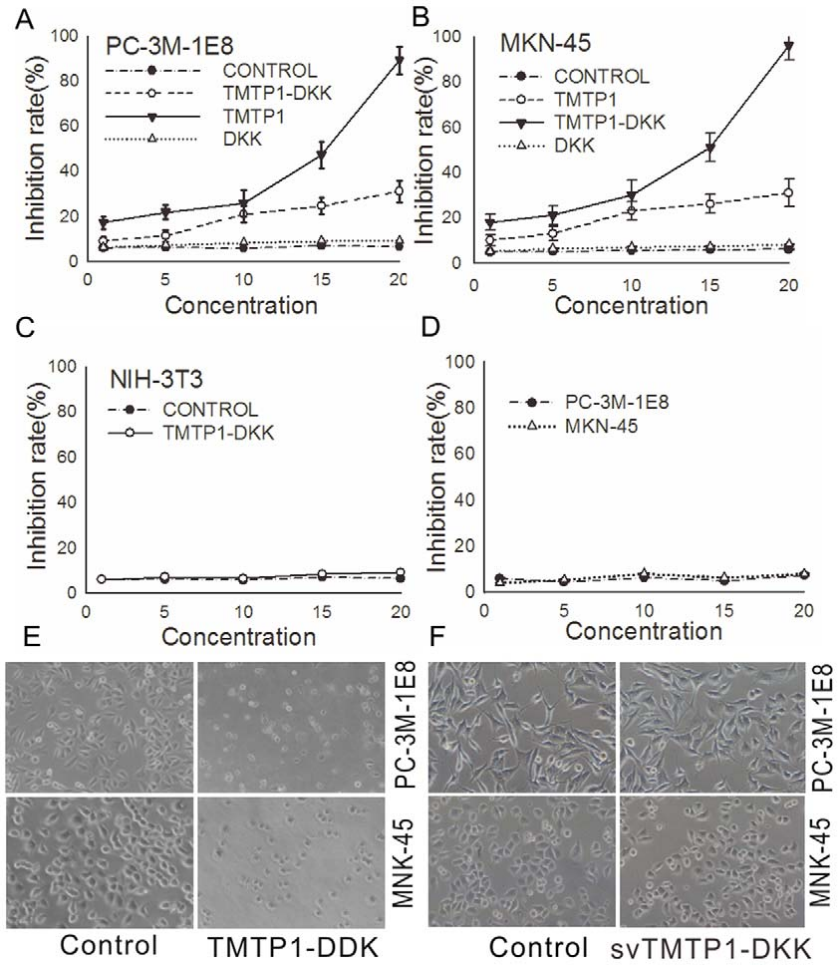
Cytotoxicity of the TMTP1-DKK peptide in various cell lines. A, B, C Cell survival rates were determined by MTT assays performed in triplicate (*error bars*, ±SD). The data presented represent the percentage of cells surviving compared to untreated cells. Representative results are shown. The differences of survival rates between 10 µM and 20 µM TMTP1-DKK are more significant in either PC-3M-1E8 or MKN-45sci cells (*P*<0.01). However, little or no effect was seen on murine fibroblast NIH/3T3 cell proliferation when they were treated with TMTP1-DKK. D Cells viability of MKN-45 and PC-3M-1E8 cancer cells treated different concentrations (0–20 µM) of svTMTP1-DKK for 24 hour was measured by MTT assay. E Morphological quantification of cellular apoptosis by inverted microscope in PC-3M-1E8 and MKN-45sci cells treated with 10 µM TMTP1-DKK. After treated with DKK, the cells showed cell shrinkage, membrane disintegration, and nuclear condensation/fragmentation. F Little Morphological change was observed by inverted microscope in PC-3M-1E8 and MKN-45sci treated with 10 µM svTMTP1-DKK for 24 hour.

We next sought to determine if TMTP1-DKK can inhibit the proliferation of highly metastatic cancer cells. To test this, PC-3M-1E8, MKN-45sci, and NIH/3T3 cells were treated with various concentrations (1–20 µM) of TMTP1-DKK and control peptides for 24 h. The rates of cell growth were measured by MTT assay. TMTP1-DKK inhibited cell proliferation of PC-3M-1E8 and MKN-45sci cells in a dose-dependent manner with the maximum inhibition occurring at a dose of 15–20 µM of TMTP1-DKK (Figure 2A and B *P*<0.01. Additionally, the inhibition rate by TMTP1 was much lower than by TMTP1-DKK (Figure 2A and B, *P*<0.01). Inhibition on the proliferation of murine fibroblast NIH/3T3 cells was not detected (Figure 2C). And svTMTP1-DKK showed no effect on the prostate and gastric cancer cells (Figure 2D, F).

**Figure 3:**
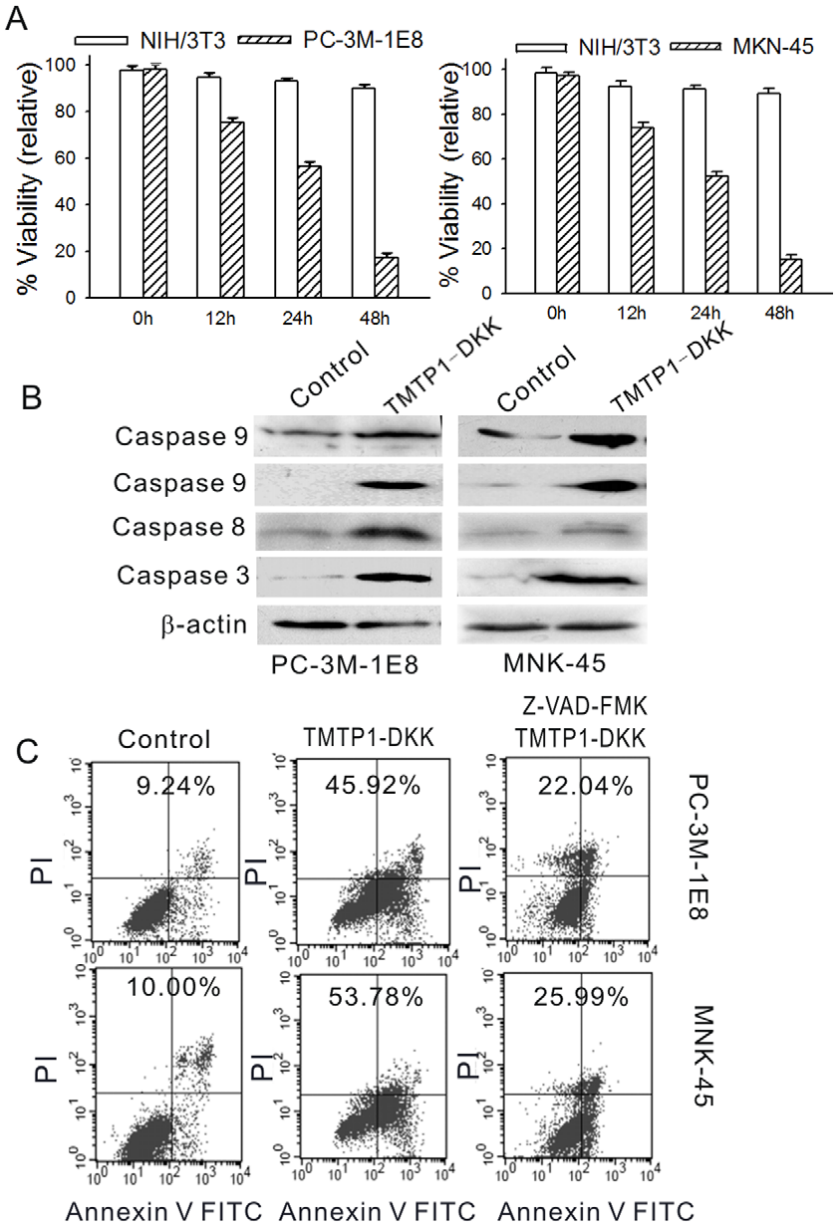
TMTP1-DKK induces apoptosis in cells. A Cells were treated overnight with 10 µM TMTP1-DKK peptide and then analyzed by flow cytometry at 12 h, 24 h, and 48 h. Proliferating PC-3M-1E8 cells and MKN-45sci cells treated with TMTP1-DKK peptide (filled bars) showed a decrease in viability through apoptosis over time (*P*<0.01). However, murine fibroblast NIH/3T3 cells showed no significant changes. **B** Signaling molecules involved in the apoptotic effect of TMTP1-DKK peptide treated. Cultured cells treated with 10 µM TMTP1-DKK peptide for 24 h were harvested and lysed. Total cell proteins were resolved by 12% SDS-PAGE gel elctrophoresis and then transferred to a nitrocellulose membrane. After blocking with 5% nonfat milk, the membranes were incubated for 1 h with 1 µg/ml primary antibodies (caspase 9 (35 KD, 17 KD), caspase 8 (20 KD) and caspase 3(19 KD)) followed by horseradish peroxidase-conjugated anti-rabbit secondary antibodies. Blots were exposed to chemiluminescence substrate and developed with Hyperfilm MP. **C** PC-3M-1E8 cells and MKN-45sci cells were challenged with 10 µM TMTP1-DKK in the presence (+) of 40 µM Z-VAD-fmk for 24 h. After 24 hours, cell viability was analyzed. The broad range caspase inhibitor Z-VAD-fmk decreased the apoptosis rate of PC-3 M-1E8 and MKN-45sci cells.

It is possible that TMTP1-DKK restricted proliferation by inducing apoptosis in highly metastatic cancer cells. To test this, PC-3M-1E8, MKN-45sci cells, normal mammary epithelial cell MCF-10A, normal liver cell LO2 and HEK293 cells were treated with 10 µM TMTP1-DKK and svTMTP1-DKK for 24 h. Any morphological changes in the cells were observed under phase-contrast microscope. Rates of apoptosis rates were determined by FACS using FITC-annexin V and propidium iodide staining. PC-3M-1E8 and MKN-45sci cells treated with TMTP1-DKK showed cell shrinkage, membrane disintegration, and nuclear condensation/fragmentation (Figure 2E), which are all characteristic morphological changes of cells undergoing apoptosis. While TMTP1-DKK did not affect these normal cells' viability and morphology under phase-contrast microscope (Figure 1B). FACS analysis also indicated that TMTP1-DKK induced cell apoptosis in a dose-dependent manner at doses of 10 µM or higher, compared with control (Figure 3A and C).

### TMTP1-DKK activates both mitochondrial and Fas-dependent apoptosis pathways

_D_(KLAKLAK)^2^ is an amphipathic D-amino acid peptide that binds selectively to bacterial, but not eukaryotic, cell membranes [9]. In eukaryotes, it initiates apoptosis through disruption of the mitochondria, presumably since the membranes of mitochondria resemble those of bacteria [10]. There are two major signaling pathways for apoptosis: the extracellular Fas death receptor pathway and the intracellular mitochondrial pathway. To identify the apoptotic pathway triggered by TMTP1-DKK treatment in PC-3M-1E8 and MKN-45sci cells, we examined the expression of caspase-3, 8, and 9 by western blotting. Bands corresponding to active caspase 3 (19 kDa), caspase 8 (20 kDa), and caspase 9 (two bands: 17 kDa and 35 kDa) were detected. Thus, in TMTP1-DKK treated cancer cells, cleavage of caspases 3, 8, and 9 were all detected. This indicated that both the Fas death receptor pathway and the mitochondrial pathway were activated by TMTP1-DKK (Figure 3B).

To further validate that TMTP1-DKK promoted apoptosis, we tested whether caspase inhibitors could decrease the TMTP1-DKK induced apoptosis rate in PC-3M-1E8 and MKN-45sci cells lines. The broad range caspase inhibitor Z-VAD-FMK could decrease apoptotic rate of TMTP1-DKK-treated PC-3 M-1E8 and MKN-45sci cells from 45.2±2.3% to 22.1±1.3% and 53.7±2.6% to 25±0.8% respectively (Figure 3C). These results suggest that TMTP1-DKK induced apoptosis through both the intracellular mitochondrial pathway and the Fas death receptor pathway.

### TMTP1-DKK inhibits invasion of tumor *in vitro*

**Figure 4:**
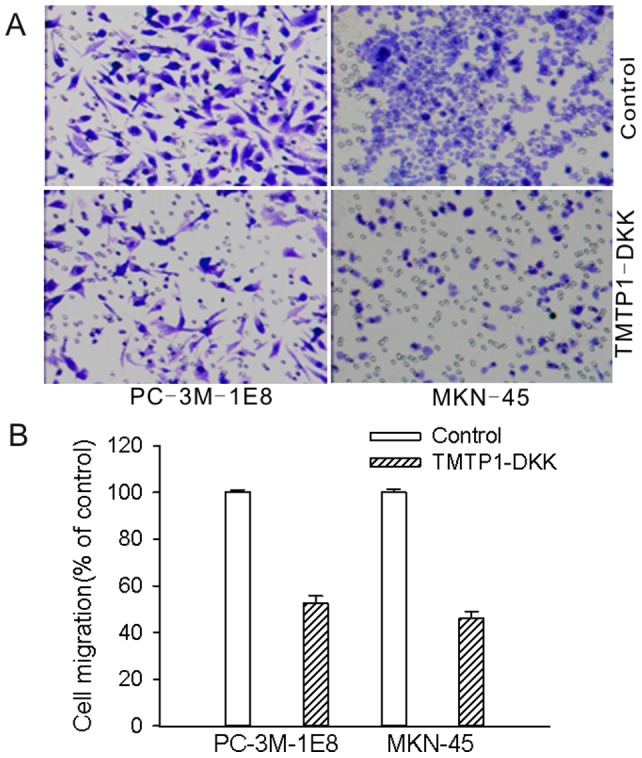
TMTP1-DKK inhibits cell migration. A Cells were incubated with 2 µM TMTP1-DKK peptide for 12 h. Transwell migration assays of PC-3M-1E8 cells and MKN-45sci cells were performed. After 24 h incubation, cells from the upper side of the filter were removed and cells from the lower surface of the filter were fixed and stained. Data are the means ± SE of three independent experiments; each performed in triplicate. **B** Cellular migration was reduced by 52.38±3.3% in PC-3M-1E8 cells and 46.16±2.7% in MKN-45sci cells compared to the appropriate controls.

To examine whether TMTP1-DKK affected cells invasion, PC-3M-1E8 and MKN-45sci cells were treated with either TMTP1-DKK or filter-sterile water for 12 h, afterward which cell invasion was determined by a matrigel invasion assay (Figure 4A). The cellular migration rate of PC-3M-1E8 cells decreased by 52.38±3.3% when cells were treated with 2 µM of TMTP1-DKK peptide. Similarly, cellular migration of MKN-45sci cells decreased by 46.16±2.7% under the same conditions (Figure 4B).

### TMTP1-DKK inhibits tumor growth and development *in vivo*

We next sought to explore if TMTP1-DKK peptide might represent a possible therapeutic strategy to suppress tumor growth *in vivo*. To test this, we analyzed the effect of TMTP1-DKK on subcutaneous tumor growth in mice. MKN-45sci orthotopic gastric cancer and PC-3M-1E8 prostate cancer cells were injected subcutaneously into mice that were then treated with 50 µM TMTP1-DKK, TMTP1 or filter-sterile water through intraperitoneal (IP) injection every other day. PC-3M-1E8 tumor–bearing mice treated with TMTP1-DKK showed an increase in survival rate from 55 days (range from 49 to 58 days) to 65 days (range from 60 to 69 days) as compared to control groups. Meanwhile, the survival time of MKN-45sci tumor-bearing mice increased from 35 days (range from 29 to 39 days) to 42 days (range from 35 to 44 days) when treated with TMTP1-DKK.

**Figure 5:**
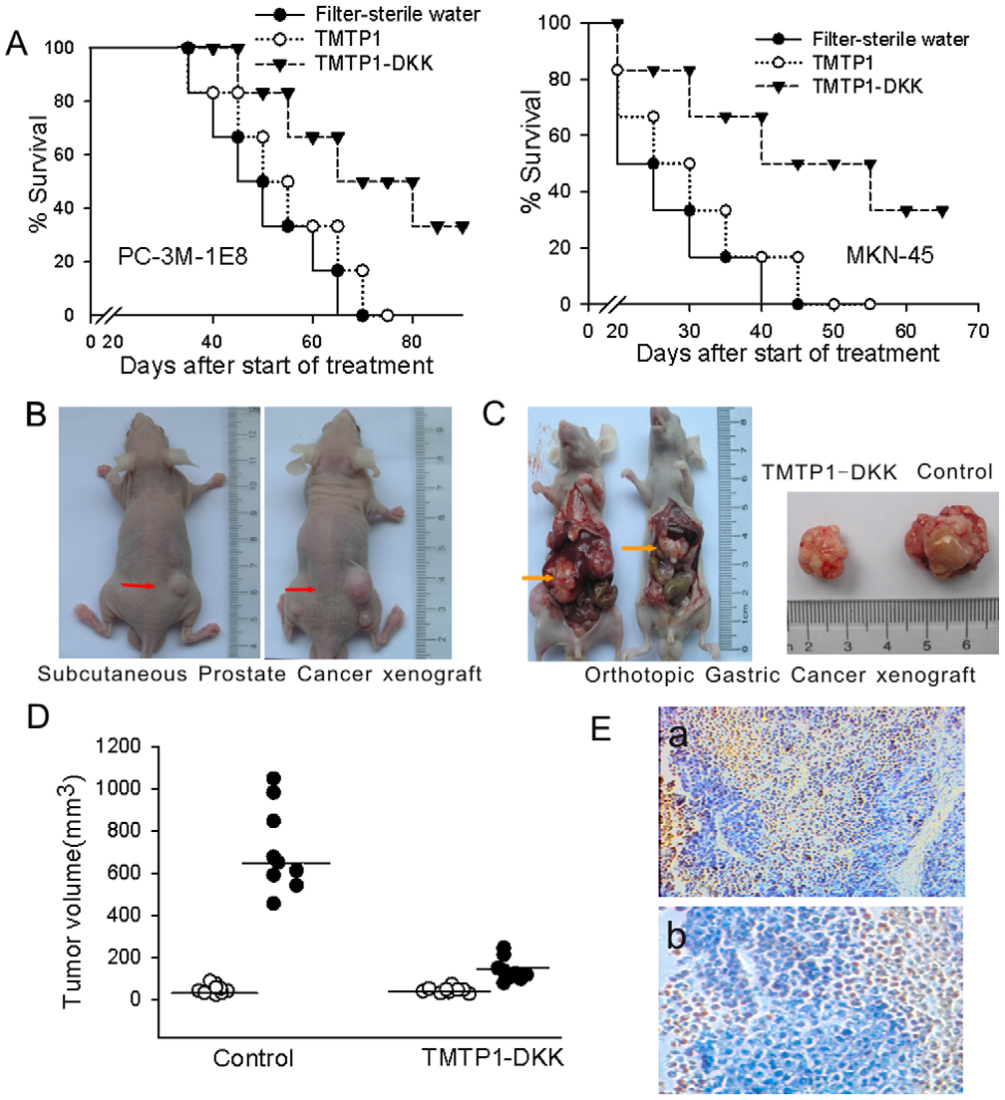
TMTP1-DKK inhibits tumor growth and development by the mouse models of MKN-45sci orthotopic gastric cancer and PC-3M-1E8 prostate cancer. **A** Mice treated with TMTP1-DKK peptide survived longer than control mice treated with an equimolar mixture of TMTP1 peptide or filter-sterile water, as shown by a Kaplan-Meier survival plot. **B, C** PC-3M-1E8 prostate cancer and MKN-45sci orthotopic gastric cancer treated with TMTP1-DKK peptide were smaller than those treated with control peptide. **D** Tumor growth in PC-3M-1E8 tumor-bearing mice undergoing TMTP1-DKK peptide therapy. Mice were treated over a period of 40 days (*n* = 9). During the treatment period, tumors were measured twice a week. Tumors volume in mice treated with TMTP1-DKK peptide was on average 18% the size of control. Tumor volumes were assessed on day 1 (○) and day 40 (•). P<0.05, t-test. **E** Animals were sacrificed six days after TMTP1-DKK treatment. The tumor was recovered and imaged immediately following analysis of apoptotic cell death by TUNEL assay. The number of apoptotic cells increased notably in TMTP1-DKK peptide–treated tumors. a, Magnification: ×100. b Magnification: ×200.

TMTP1-DKK treatment also markedly inhibited tumor growth in PC-3M-1E8 subcutaneous prostate cancer and MKN-45sci orthotopic gastric cancer bearing mice. The mean volume of both types of xenograft tumors shrunk in the TMTP1-DKK treated group relative to control (Figure 5B, C). Forty days after injection, the PC-3M-1E8 subcutaneous prostate cancer xenograft tumor volume was on average 18% that of control in TMTP1-DKK treated mice (Figure 5D). Furthermore, we investigated whether TMTP1-DKK peptide induced apoptosis in the xenograft prostate tumors *in vivo* by TUNEL assay. The number of observed apoptotic cells increased in TMTP1-DKK peptide treated group relative to control. Thus, these results show a vigorous anti-tumor effect of TMTP1-DKK peptide *in vivo*.

### TMTP1-DKK suppresses tumor metastasis and progression of MKN-45sci orthotopic xenografts in athymic mice

**Figure 6:**
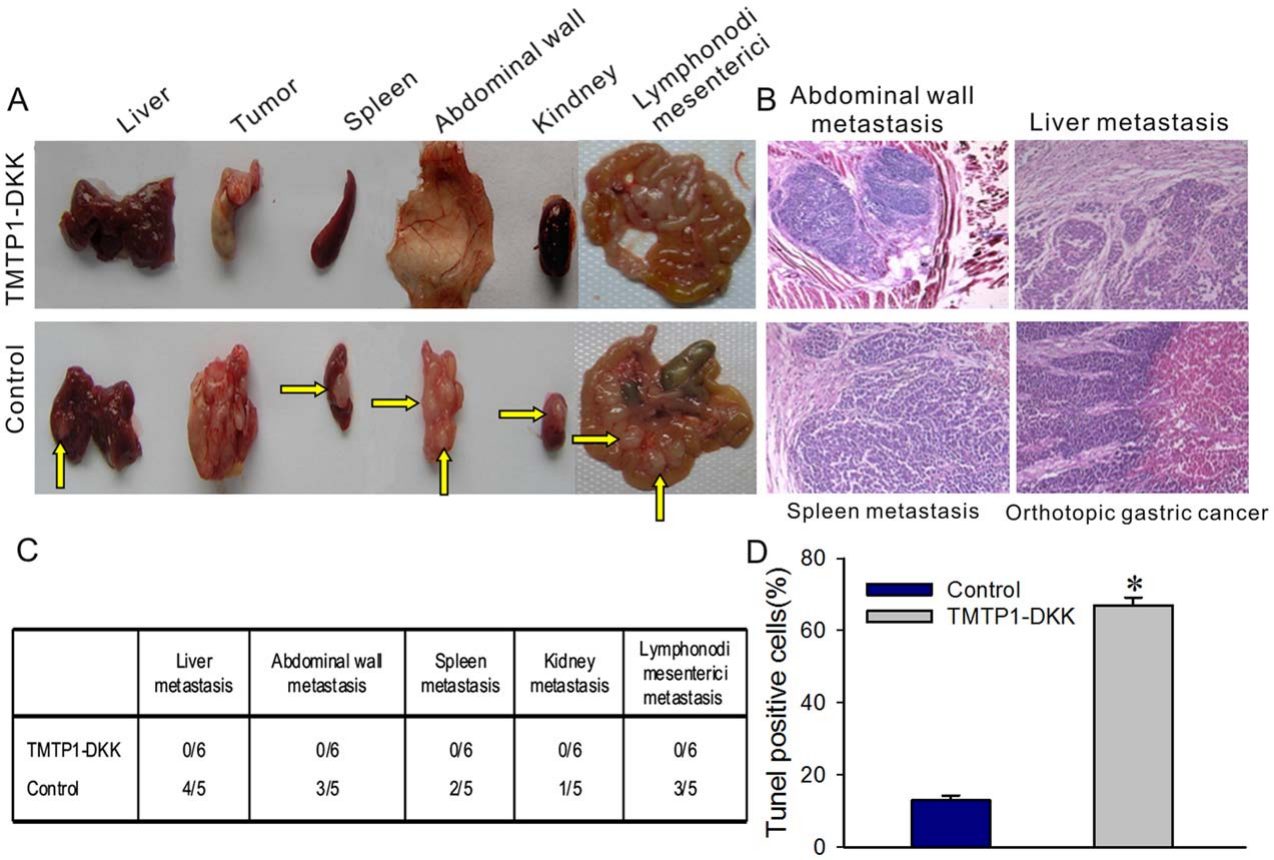
TMTP1-DKK suppresses tumor metastasis and progression by MKN-45sci orthotopic xenografts in athymic mice. A, B TMTP1-DKK peptide caused a dramatic and dose-dependent decrease in the number of metastatic tumors (yellow arrows) in MKN-45sci orthotopic gastric cancer. **C** Metastasis tissue samples were harvested and pathologically confirmed by H&E staining. **D** Orthotopic gastric tumors were recovered and imaged immediately following analysis of apoptotic cell death by TUNEL assay. Columns, average number of TUNEL-positive cells counted in three randomly selected fields in three tumor samples from each group; error bars, SD. *, P<0.001 by Student's t test compared with the control group.

To test if TMTP1-DKK can decrease metastasis *in vivo*, we examined the effects of this peptide in a metastasis model. Orthotopic implantation of human gastric cancer MKN-45sci fragments into the stomach of six nude mice caused a significant increase in the number of metastatic tissues, including four liver metastasis, two spleen metastasis, three abdominal wall metastasis, one kidney metastasis, and two mesenteric lymph node metastasis (one control mice died within 16 days of implantation). However, IP administration of TMTP1-DKK every other day for 20 days caused a dramatic and dose-dependent decrease in the number of metastatic tissues, with no metastasis being observed in six TMTP1-DKK treated nude mice (Figure 6A, C). In addition, H&E-safranin analyses of liver metastasis, spleen metastasis, abdominal wall metastasis, and kidney metastasis revealed that development of the stage of metastatic foci (Figure 6B). Thus, we conclude that TMTP1-DKK treatment significantly decreased tumor growth and metastasis *in vivo*. Furthermore, apoptosis of cells in the orthotopic gastric tumor was detected by TUNEL assay (For quantification, see Figure 6D).

TMTP1-DKK-treated mice survived well and showed no signs of toxicity or body weight loss throughout the experiments. Collectively, these results suggest that TMTP1-DKK can inhibit tumor metastasis and tumor growth *in vivo*.

## Discussion

The treatment of cancer using micromolecular therapeutics such as oligonucleotides or peptides requires efficient delivery systems capable of intracellular penetration. Recently, a series of novel peptides were identified that bind specifically to the plasma membrane of both cancer and tumor-associated endothelial cells. The identified peptides were promising alternatives to currently used biomolecules for targeting metastatic cells due to their rapid blood clearance, increased diffusion and tissue penetration, nonimmunogenic nature, and ease of synthesis [21], [22], [23], [24], [25]. We have previously demonstrated that occult metastases or subclinical micrometastases were distinctively detected by TMTP1 [11]. In the present study, we further investigated whether TMTP1 could deliver therapeutic agents efficiently. We examined the ability of TMTP1 to deliver a proapoptotic peptide to highly metastatic cancer cells by coupling it to the pro-apoptotic _D_(KLAKLAK)^2^ domains. We chose the synthetic 14-amino-acid peptide KLAKLAKKLAKLAK because it was inert outside of cells but became toxic when internalized causing disruption of the mitochondrial membrane, leading to programmed cell death.

Our data shows that TMTP1-GG-_D_(KLAKLAK)^2^ bound specifically to highly metastatic tumor cell lines, prostate cancer cell PC-3M-1E8 and gastric cancer cell MKN-45sci *in vitro* and *in vivo*. However, TMPT1-GG-_D_(KLAKLAK)^2^ did not bind to the nometastatic prostate cancer cell PC-3M-2B4 and murine fibroblast cell line NIH/3T3 normal mammary epithelial cell MCF-10A, normal liver cell LO2 and HEK293 cells (Figure 1). Results showed that TMTP1-DKK did not affect these normal cells' viability and morphology. Since we observed FITC-TMTP1-GG-_D_(KLAKLAK)^2^ binding to both cell lines and xenograft tumors in nude mice, we reasoned that TMTP1 would target delivery of the pro-apoptotic peptide to highly metastatic tumors.

We further examined the anti-tumor effects of the TMTP1-DKK fusion peptide. Our results show that TMTP1-DKK mediated remarkable anti-tumor activities both i*n vitro* and *in vivo*, compared to either TMTP1 or DKK alone. The fusion peptide TMTP1-DKK induced apoptosis rapidly and potently. Low concentrations of TMTP1-DKK could induce cancer cell death while having little effect on the murine fibroblast cells NIH/3T3. TMTP1-DKK showed high anti-tumor effects on highly metastatic MKN-45sci and PC-3M-1E8 cells, compared to TMTP1. DKK alone could not trigger apoptosis as it could not be transduced to the cytoplasm of tumor cells. When linked to the targeting peptide TMTP1, micromolar levels of DKK resulted in at least 100-fold increase of killing efficiency in cancer cells. Interestingly, the increased killing efficiency was not observed in the murine fibroblast cell line NIH/3T3. Moreover, TMTP1-DKK significantly inhibited the invasiveness of tumor cells *in vitro* in a transwell assay. These data also demonstrate the modular nature of the targeting/transduction domain and the pro-apoptosis domain in TMTP1-DKK. Each domain confers its properties upon the coupled peptide to generate a biologically active agent.

_D_(KLAKLAK)^2^ has antibacterial activity but is relatively nontoxic to eukaryotic cells. However, it has been shown that if _D_(KLAKLAK)^2^ is delivered into the cytoplasm of mammalian cells, it disrupts mitochondria, due to similarity of mitochonrdrial and bacterial membranes, and initiates apoptosis [26]. Previous studies showed that when _D_(KLAKLAK)^2^ was conjugated to a homing peptide through a G-G linker that it homes to tumor vasculature and was selectively cytotoxic to angiogenic endothelial cells and had anti-tumor activity *in vivo* [26]. Following internalization, the proapoptotic _D_(KLAKLAK)^2^ peptide acted by breaking down mitochondrial membrane, which causes cytochrome c efflux into the cytoplasm resulting in apoptosis. In the cytoplasm, cytochrome c forms a complex with Apaf-1 and procaspase-9. Interaction with procaspase-9 results in its cleavage and activation, initiating cleavage of downstream effector caspases. In our study, we observed activated caspase 9 by western blot in TMTP1-DKK treated tumor cells, suggesting involvement of the mitochondrial pathway (Figure 3). We also observed activation of caspase 8, which suggested the Fas-ligand extracellular cell death pathway was involved in TMTP1-DKK induced apoptosis as well. The efficiency of TMTP1-DKK induced apoptosis was inhibited by the broad range caspase inhibitor Z-VAD-fmk in PC-3M-1E8 and MKN-45sci cells. Therefore, our study demonstrated that TMTP1-DKK triggered apoptosis through both the mitochondrial pathway and the death receptor pathway.

TMTP1-DKK appeared to selectively inhibit tumor growth and have little effect on other tissues in the subcutaneous xenograft and orthotopic transplantation mice models. Both the subcutaneous xenograft tumors of PC-3M-1E8 and the murine MKN-45sci orthotopic gastric tumors were remarkably inhibited by TMTP1-DKK. The survival time of tumor bearing nude mice in both models was significantly prolonged by TMTP1-DKK treatment. Cessation of therapeutic peptide administration led to rapid tumor growth and death of experimental animals. Furthermore, we provided direct experimental evidence in a preclinical model of the efficacy of interference of orthotopic growth of human gastric cancer cells in nude mice at both the primary site and metastases. We observed tumor metastases in all untreated animals, but only a few in TMTP1-DKK treated animals. Thus, the results from the orthotopic MKN-45sci xenografts revealed that TMTP1-DKK efficiently inhibited tumor metastasis in mice. The therapeutic effects of TMTP1-DKK peptide *in vivo* were also shown to be the result of tumor cells apoptosis by as demonstrated by a TUNEL assay (Figure 5, 6).

Taken together, these data suggest that TMTP1-DKK is an efficient anti-tumor agent both *in vitro* and *in vivo*. It triggered apoptosis in a series of highly metastatic cancer cells via both the mitochondrial pathway and death receptor pathway. Our results suggest that TMTP1-DKK may be a powerful candidate therapeutic agent for metastatic tumors based on its specific targeting and effective tumor-killing activity. Further study of TMTP1-DKK and its analogues may lead to the discovery of new anti-tumor and metastasis agents.

## Materials and Methods

### Peptide design and synthesis

TMTP1-DKK (NVVRQ -GG-_D_(KLAKLAK)^2^) and svTMTP1-DKK(VNQRV -GG-_D_(KLAKLAK)^2^), which is the control peptide of TMTP1 (NVVRQ), were created by coupling the targeting domain (TMTP1) and the pro-apoptotic _D_(KLAKLAK)^2^ domain through a glycinylglycine bridge as described [11]. Fluorescein isothiocyanate (FITC) was coupled to the peptide via an additional glycine at the N-terminus. TMTP1-DKK and control peptides (DKK, TMTP1 and svTMTP1-DKK) were synthesized using Fmoc chemistry in a solid-phase synthesizer and purified by HPLC by Xi'an Huachen Bio-tech Ltd (Xi'an, China). The sequence and structure of the peptides were confirmed by mass spectrometry. Peptides were dissolved in filter-sterile water to a concentration of 1 mM.

### Cell lines and Reagents

The highly metastatic and nonmetastatic human prostate cancer cell lines PC-3M-1E8 and PC-3M-2B4 respectively were kindly provided by Dr. Jie Zheng (Peking University, Beijing, China) [27], [28]. The human gastric cancer cell lineMKN-45sci, the murine fibroblast NIH/3T3 cells, normal mammary epithelial cell MCF-10A, normal liver cell LO2 and HEK293 cells were obtained from the American Type Culture Collection (Manassas, VA, USA). All cells were maintained in RPMI 1640 supplemented with 10% fetal calf serum (FCS) at 37 °C with 5% CO_2_. Z-VAD-FMK was obtained from Bachem (Heidelberg, Germany).

### Construction of mouse models

Four-week-old BALB/c *nu/nu* mice were obtained from the SLAC Laboratory Animal Co. Ltd (Shanghai, China). In the direct intrathecal (IT) injection studies, 3×10^6^ PC3M-1E8 cells were suspended in 100 μl normal saline and injected subcutaneously (SC) in the right flanks of mice (4–6 weeks old). Tumors were allowed to grow to 4–6 mm in diameter before treatment. Fresh tumor fragments (2 mm^3^) were then implanted SC into the posterior trunk of the anesthetized mice.

The mouse model of MKN-45sci orthotopic gastric cancer, which has the potential for liver-specific metastasis, was kindly provided by Dr. Jinjun Li (Shanghai Cancer Institute, Medical College of Shanghai Jiao Tong University, Shanghai, China) [29], [30], [31]. Fresh tumor fragments were obtained as described above. After mouse anesthetization, the stomach was exposed and the part of the serosal membrane scraped with forceps. One 1 mm^3^ tumor piece was then fixed on the scraped site of the serosal surface with a 5–0 absorbent suture. The stomach was then returned to the peritoneal cavity, and the abdominal wall and skin were closed with 1–0 sutures.

Animal experiments were approved by the Hubei Institute Animal Research Committee. All animals were bred at our animal facility according to the Chinese Laboratory Animal Guidelines.

### Peptide *in vitro* binding and internalization experiments

Cells were seeded onto glass coverslips and cultivated for 24 h until 60% confluence. The medium was replaced with 1 ml fresh medium supplemented with 10% FCS and 1 µM FITC-conjugated peptide. Cells were then cultivated for 2 h. The cells were washed with PBS 3 times and then fixed with methanol/acetone (1∶1). The nuclei of tumor cells were visualized by 4,6-diamidino-2-phenylindole (DAPI) staining. Cells were examined under a fluorescence microscope.

### Tumor targeting

Tumor-bearing mice were used for homing experiments after the tumors had grown to a size of 1.0–1.5 cm^3^. FITC-conjugated peptide (300 µg in 50 µl filter-sterile water) was injected into the tail vein and allowed to circulate for 48 h. The mice were then anesthetized and perfused with 5 ml PBS through the left ventricle at the indicated time points. Tumors and control organs including heart, liver, spleen, lung, kidney, brain, prostate, and small intestine were removed, frozen in OCT embedding medium (Tissue-Tek, Elkhart, U.S.A.), sliced, and examined for fluorescence by laser scanning confocal microscopy (Olympus Fluoview FV1000, Japan). The nuclei of tumor cells were visualized by DAPI staining.

### Cell Growth Assay

Cell growth was measured using the 3–(4, 5 dimethylthiazol-2yl)-2, 5-diphenyl-tetrazolium bromide (MTT; Sigma) colorimetric dye method as previously described [32]. Briefly, PC-3M-1E8, MKN-45sci and NIH/3T3 cells were plated overnight at 5000 cells per well in 96-well plates. Cells were then treated with 1 µM, 5 µM, 10 µM, 15 µM, or 20 µM of TMTP1-DKK or control peptides for 24 hours. The inhibition of the cell growth was measured and calculated according to the formula: inhibition rate (%)  =  (control value A490-experimental value A490)/control value A490×100%.

### Apoptosis Assay

PC-3M-1E8, MKN-45sci, NIH/3T3 cells, normal mammary epithelial cell MCF-10A, normal liver cell LO2 and HEK293 cells that were 80% confluent were treated with 10 µM TMTP1-DKK in the presence of 40 µM Z-VAD-FMK for 24 h. Cells were observed under an inverted microscope (Nikon TE 300), harvested in 5 mM EDTA in PBS, washed and resuspended in annexin-binding buffer, and then stained with annexin V and propidium iodide according to manufacturer's instructions (Calbiochem, San Diego, CA). Apoptotic cells were analyzed by FACS Calibur (BD Biosciences). Experiments were conducted in triplicate.

### Western blotting analysis

Cells pre-treated with 10 µM TMTP1-DKK peptide for 24 h were harvested and lysed. Total cell proteins were resolved by 12% SDS-PAGE, transferred to polyvinylidene difluoride membranes (90 V for 2 h), and probed with antibodies directed against human caspase-3 and caspase-9 (Cell Signaling Technology,), caspase-8 (Lab Vision & NEOMARKERS), and β-actin (Santa Cruz Biotechnology). Horseradish peroxidase conjugated goat anti-rabbit antibody was used as the secondary antibody. Proteins were visualized with chemiluminescence reagents (Santa Cruz Biotechnology). Protein bands were scanned with a densitometer and their relative intensities quantified using ImageQuant software (Molecular Dynamics, Sunnyvale, CA).

### Invasion assay

The cell invasion was investigated using the transwell system (6.5 mm in diameter with 8 µm pore size). PC-3M-1E8 and MKN-45sci cells were pre-incubated with 2 µM TMTP1-DKK peptide for 12 h. Polycarbonate membranes of the transwell system were coated with 50 µl of matrigel overnight. 1×10^5^ treated or control cells were placed on the upper chambers in 25 µl of serum-free medium. The lower chambers were filled with conditioned NIH-3T3 medium. 24 h later, the top surface of the membrane was gently scrubbed with a cotton swab. Cells that had invaded the bottom surface of the membrane were fixed in methanol and stained with trypan blue. The number of invasive cells was counted using four (200x) fields/chamber. Each sample was repeated in triplicate.

### Mouse Experimental Techniques

The mouse models of MKN-45sci orthotopic gastric cancer and PC-3M-1E8 subcutaneous prostate cancer were established as described above. Five days after tumor transplantation, 50 µM TMTP1-DKK peptide or filter-sterile water was injected intraperitoneally (IP) every other day. Tumor growth was measured in three dimensions twice a week by a caliper. Tumor volume was calculated using the formula: length × width^2^ ×0.52 [33]. TMTP1-DKK peptide treated mice were sacrificed (at what time point) and the tumors were surgically removed for TUNEL assays.

The experimental metastatic animal model of MKN-45sci orthotopic gastric cancer was established as described above. The TMTP1-DKK peptide or filter-sterile water was injected intraperitoneally every other day. Mice were sacrificed 5 days later after the final therapy. The metastatic tissues were removed and counted. Some tissues were fixed in 10% neutral-buffered formalin solution and stained with H&E and safranin. Metastatic tumors were surgically removed for TUNEL assays.

### TUNEL assay

Tumor samples were fixed in 10% buffered formalin for 24 h, processed, and embedded in paraffin for sectioning according to conventional methods. The paraffin-embedded tumor sections (5 µm thick) were heat immobilized, deparaffinized with xylene, and then rehydrated in a graded series of ethanol. TUNEL staining of the tissue sections were performed with the In Situ Cell Death Detection Kit (Roche, Indianapolis, IN, USA) according to the manufacturer's instructions.

### Statistical analysis

All *in vitro* experiments were repeated at least three times. The software package SPSS13.5 was used to analyze data. The two-tailed Student's t test was used for comparisons between groups. *P*<0.05 was defined as statistically significant. All values were presented as mean ± standard deviation (SD).

## Supporting Information

Figure S1 **Dynamic biodistribution of TMTP1-DKK after systemic administration in the mouse models of MKN-45sci orthotopic gastric cancer and PC-3M-1E8 subcutaneous prostate cancer.** TMTP1-DKK was accumulated in the tumor 1 hour after injection and lasted at a high level for 48 h.
(TIF)

Figure S2 **Distribution of TMTP1-DKK in PC-3M-1E8 tumor-bearing mice.** The targeting assay was done as mentioned in Materials and Methods. The distribution of FITC-conjugated TMTP1-DKK in dissected tumor (*A*) and organs, including heart (*B*), liver (*C*), spleen (*D*), lung (*E*), kidney (*F*), brain (*G*), prostate (*H*), and intestine (*I*), were examined by fluorescence microscopy (Nikon TE1000-S). Magnification, ×100. The fluorescence intensity was quantified by using Image-Pro Plus 5.1 (Media Cybernetics; *J*). The autofluorescence background in mice that did not receive fluorescent compound was subtracted from the experimental values. _D_(KLAKLAK)^2^ was used as the control peptide. *Columns,* average of three independent experiments; *bars,* SD.
(TIF)
