# Expression of Concern: Anti-Tumor Effects of the Peptide TMTP1-GG-_D_(KLAKLAK)^2^ on Highly Metastatic Cancers

**DOI:** 10.1371/journal.pone.0231923

**Published:** 2020-04-14

**Authors:** The PLOS ONE Editors

After the publication of this article [1], the authors notified the journal office of an error in an image panel in Fig 2F; further concerns were identified in Fig 4A during editorial follow up. Specifically:

- In Fig 2F, the image panel representing PC-3M-1E8 cells treated with 10 μM svTMTP1-DKK is incorrect, and shares a region of overlap with the panel representing PC-3M-1E8 control cells.
- In Fig 4A, there is a region of overlap between the PC-3M-1E8 control panel and the PC-3M-1E8 TMTP1-DKK panel.

**Fig 2:**
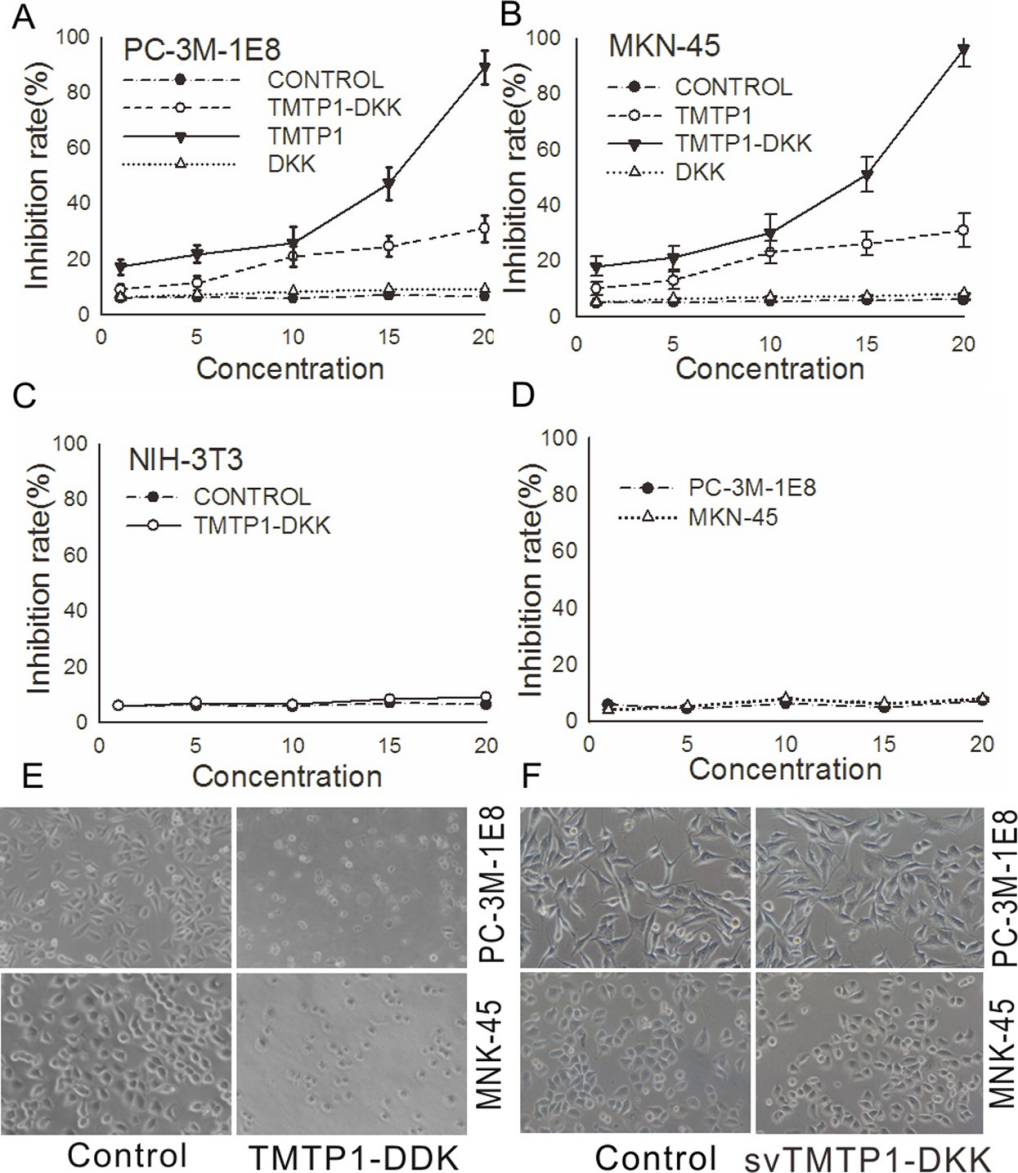
Cytotoxicity of the TMTP1-DKK peptide in various cell lines. **A, B, C** Cell survival rates were determined by MTT assays performed in triplicate (error bars, ±SD). The data presented represent the percentage of cells surviving compared to untreated cells. Representative results are shown. The differences of survival rates between 10 μM and 20 μM TMTP1-DKK are more significant in either PC-3M-1E8 or MKN-45sci cells (P<0.01). However, little or no effect was seen on murine fibroblast NIH/3T3 cell proliferation when they were treated with TMTP1-DKK. D Cells viability of MKN-45 and PC-3M-1E8 cancer cells treated different concentrations (0–20 μM) of svTMTP1-DKK for 24 hour was measured by MTT assay. E Morphological quantification of cellular apoptosis by inverted microscope in PC-3M-1E8 and MKN-45sci cells treated with 10 μM TMTP1-DKK. After treated with DKK, the cells showed cell shrinkage, membrane disintegration, and nuclear condensation/fragmentation. F Little Morphological change was observed by inverted microscope in PC-3M-1E8 and MKN-45sci treated with 10 μM svTMTP1-DKK for 24 hour.

**Fig 4:**
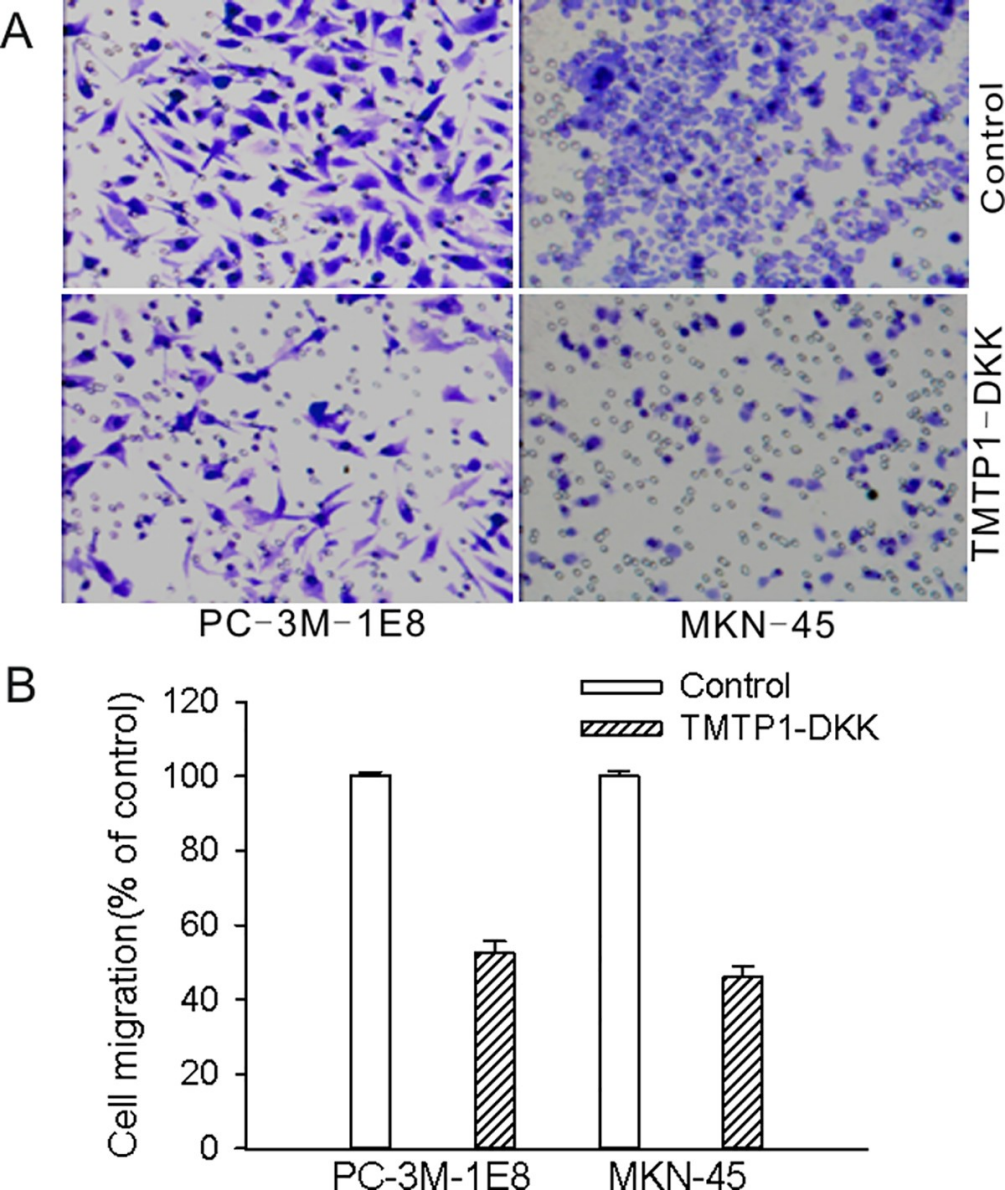
TMTP1-DKK inhibits cell migration. **A** Cells were incubated with 2 μM TMTP1-DKK peptide for 12 h. Transwell migration assays of PC-3M-1E8 cells and MKN-45sci cells were performed. After 24 h incubation, cells from the upper side of the filter were removed and cells from the lower surface of the filter were fixed and stained. Data are the means ± SE of three independent experiments; each performed in triplicate. **B** Cellular migration was reduced by 52.38±3.3% in PC-3M-1E8 cells and 46.16±2.7% in MKN-45sci cells compared to the appropriate controls.

The authors indicated that there was an error in the preparation of the figures, and that this does not affect the results and conclusions of the article. They also indicated that the original data underlying the published figures are no longer available, and they have provided results from repeat experiments for Figs 2 and 4, available via this notice as Supporting Information (S1 File, the underlying data from the repeat experiments is included as S2 File, S3 File). The cell survival rates in the repeat experiments were determined by CCK8 assays, as opposed to MMT assays used for the original Fig 2. The authors have indicated this change in methodology is due to the high sensitivity and colour stability of CCK8 assays for detecting cell proliferation

Additional information regarding the mouse experimental techniques have been provided by the authors as follows:

For assessment of survival, mice were sacrificed when tumors grew to a diameter of 2.0 cm (or 4.2 cm3) or when it was observed that an animal was in poor physical condition. The animals were monitored at least twice weekly. Endpoints for the survival study were based on the following:

- A body condition score of 1/5 based on scoring system from “1” (emaciated/wasted) to “5” (obese).
- A body condition score of 2/5 and the animal displayed a profoundly lethargic state (significantly decreased activity/responsiveness).
- The tumor affected the animal’s gait or normal posture, ability to eat, urinate, or defecate independent of tumor size.

The *PLOS ONE* Editors issue this Expression of Concern to alert readers to the errors in Figs 2 and 4, and to the concerns about the reliability of the images in those figures.

## Supporting information

S1 File Repeat experiment results. (ZIP)

S2 File Fig 2 repeat experiment data. (ZIP)

S3 File Fig 4 repeat experiment data. (ZIP)
