# Supplementary figures and images for: Anti-Tumor Effects of the Peptide TMTP1-GG-D(KLAKLAK)2 on Highly Metastatic Cancers
Source: PLoS One. 2012 Sep 11;7(9):e42685. doi: 10.1371/journal.pone.0042685 (PMC3439480; doi:10.1371/journal.pone.0042685)

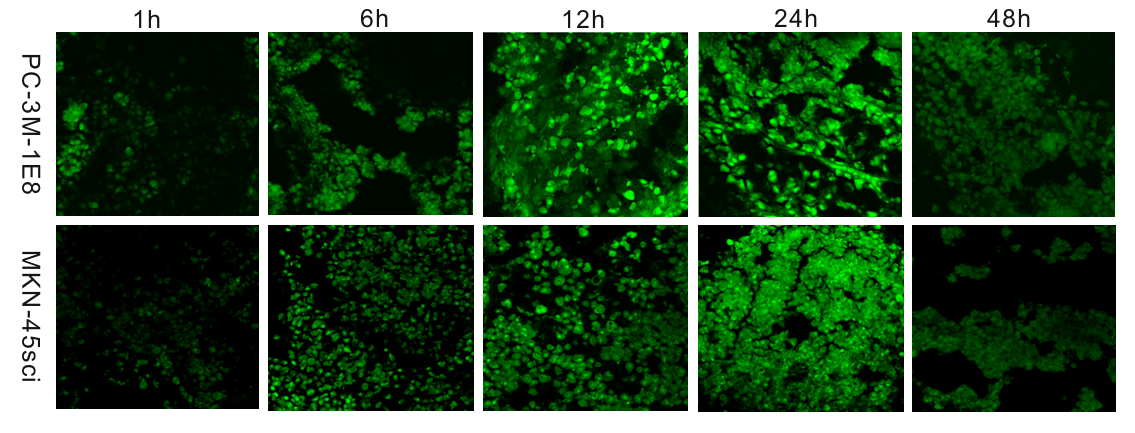

Supplement: Figure S1 — Dynamic biodistribution of TMTP1-DKK after systemic administration in the mouse models of MKN-45sci orthotopic gastric cancer and PC-3M-1E8 subcutaneous prostate cancer. TMTP1-DKK was accumulated in the tumor 1 hour after injection and lasted at a high level for 48 h. (TIF) [file pone.0042685.s001.tif]

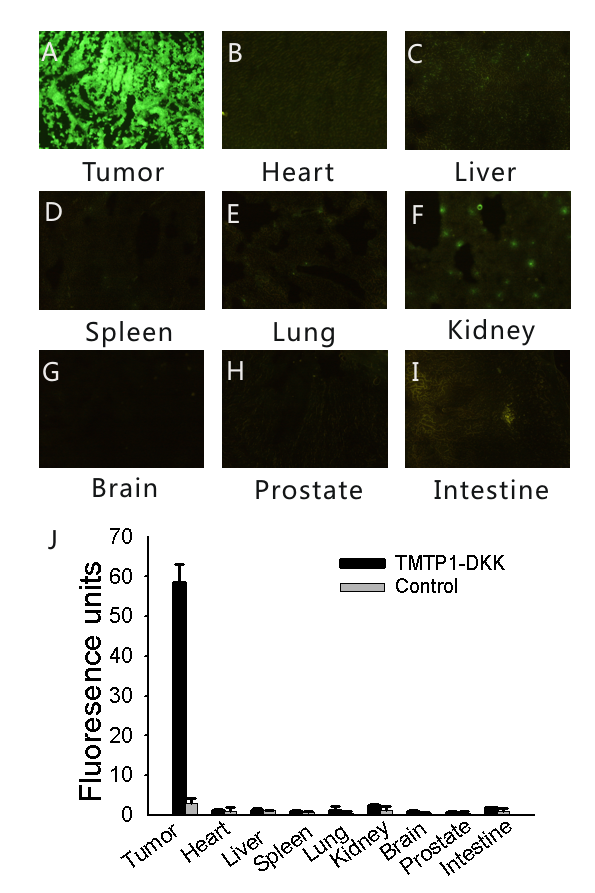

Supplement: Figure S2 — Distribution of TMTP1-DKK in PC-3M-1E8 tumor-bearing mice. The targeting assay was done as mentioned in Materials and Methods. The distribution of FITC-conjugated TMTP1-DKK in dissected tumor (A) and organs, including heart (B), liver (C), spleen (D), lung (E), kidney (F), brain (G), prostate (H), and intestine (I), were examined by fluorescence microscopy (Nikon TE1000-S). Magnification, ×100. The fluorescence intensity was quantified by using Image-Pro Plus 5.1 (Media Cybernetics; J). The autofluorescence background in mice that did not receive fluorescent compound was subtracted from the experimental values. D(KLAKLAK)2 was used as the control peptide. Columns, average of three independent experiments; bars, SD. (TIF) [file pone.0042685.s002.tif]
