# Supplementary figures and images for: Expression of Concern: Anti-Tumor Effects of the Peptide TMTP1-GG-D(KLAKLAK)2 on Highly Metastatic Cancers
Source: PLoS One. 2020 Apr 14;15(4):e0231923. doi: 10.1371/journal.pone.0231923 (PMC7156085; doi:10.1371/journal.pone.0231923)

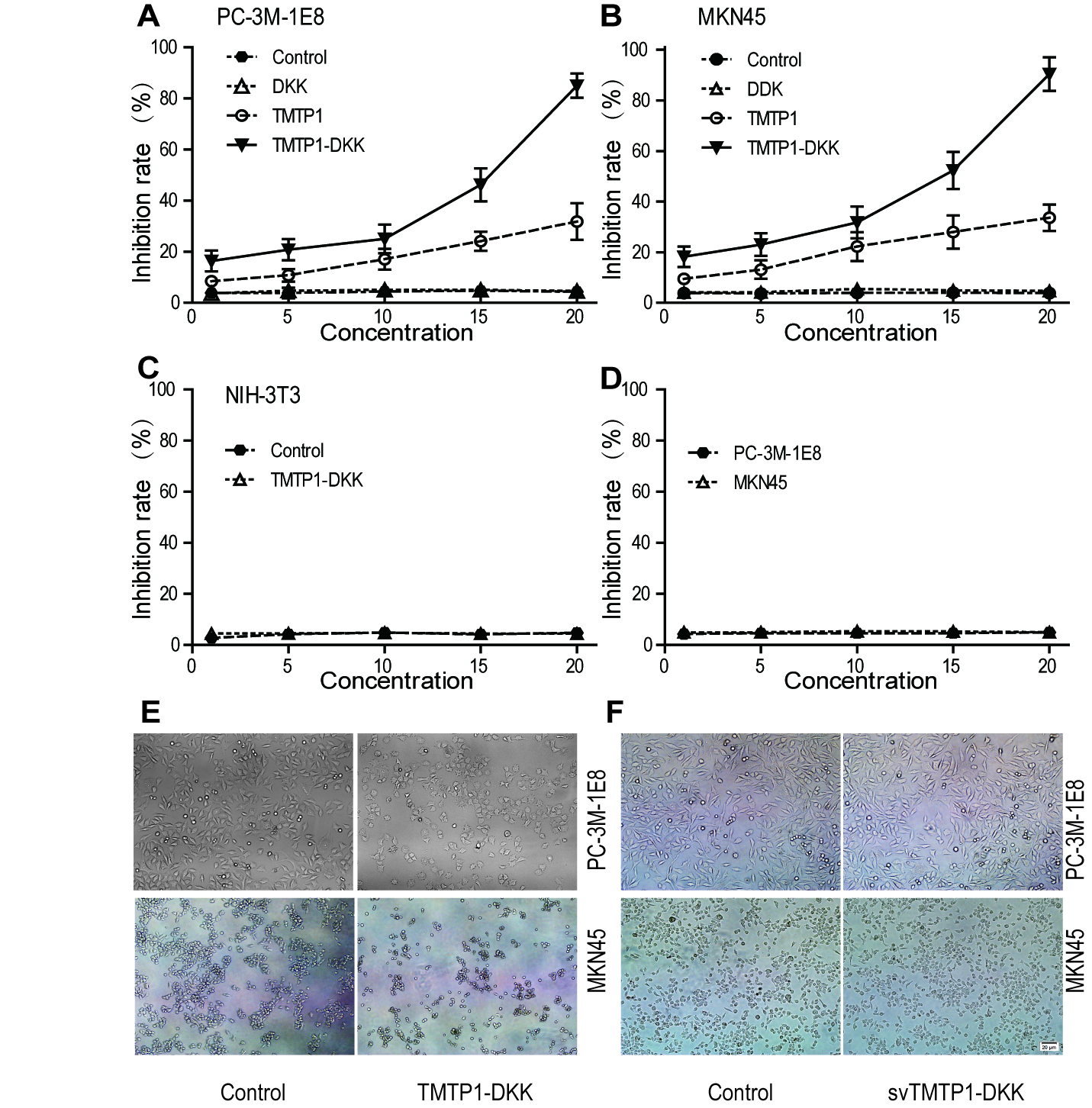

Supplement: S1 File — (ZIP) [file pone.0231923.s001.zip › Repeat Experiment Results Figure 2.tif]

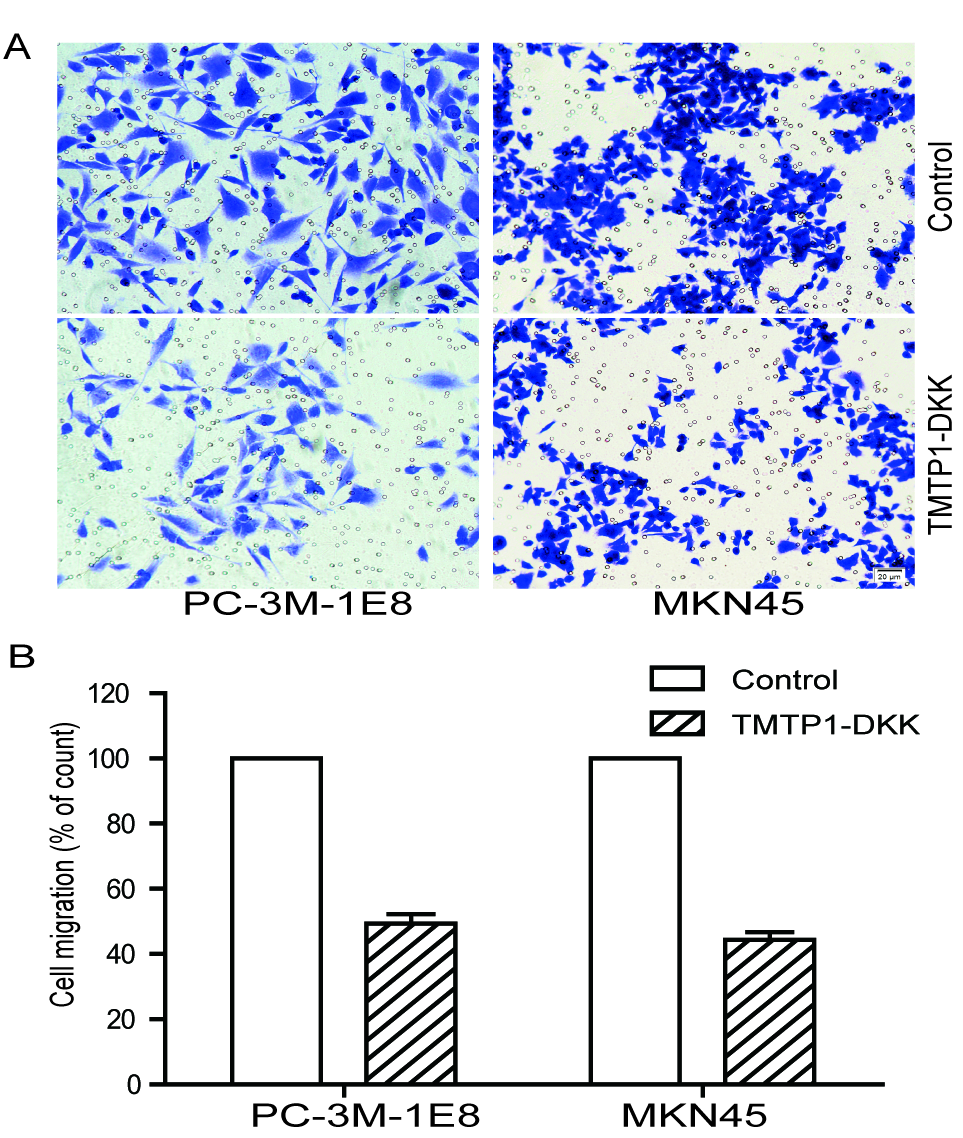

Supplement: S1 File — (ZIP) [file pone.0231923.s001.zip › Repeat Experiment Results Figure 4.tif]

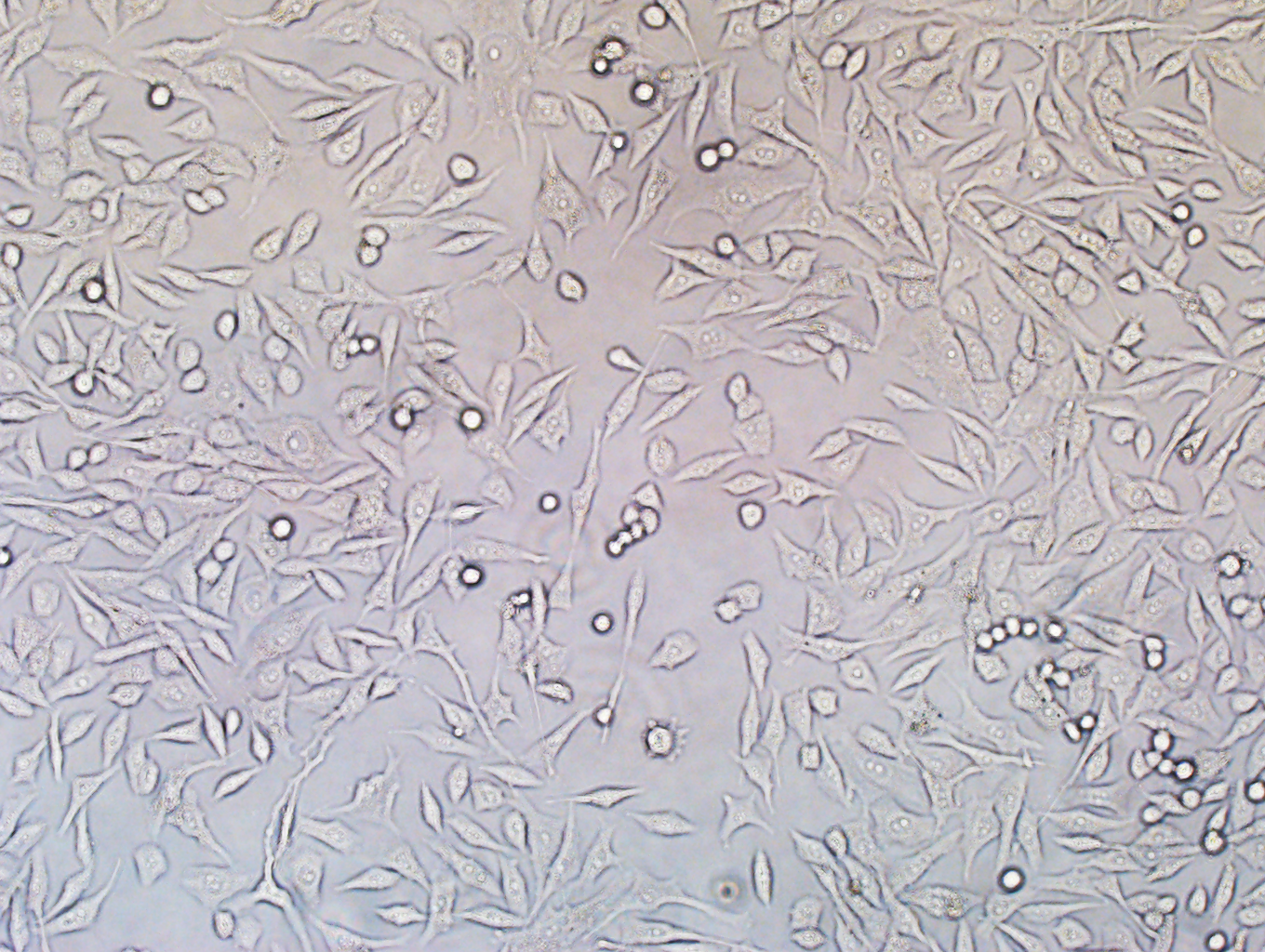

Supplement: S2 File — (ZIP) [file pone.0231923.s002.zip › Fig 2 original data/Morphological change/1E8/control/control-1.tif]

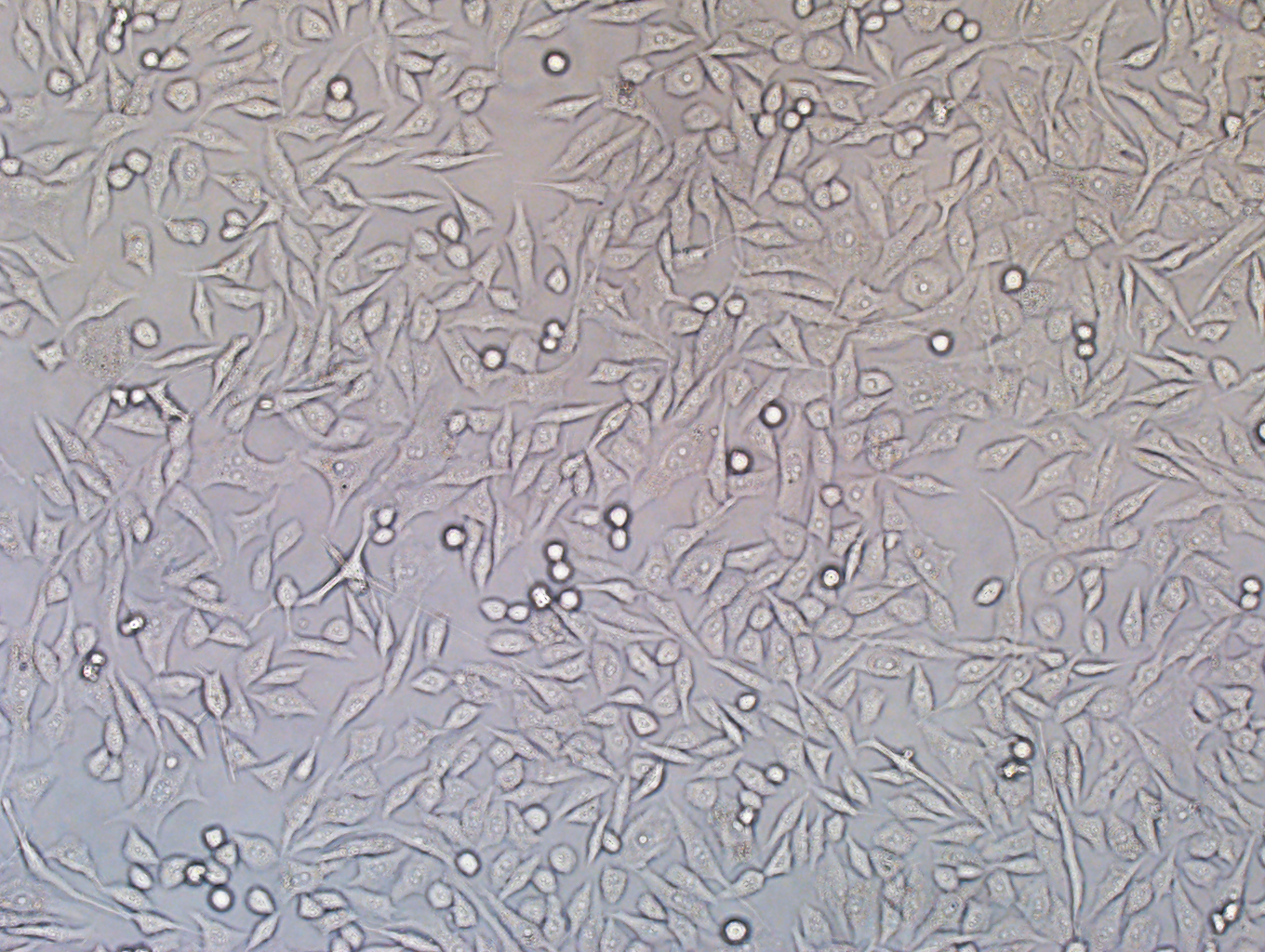

Supplement: S2 File — (ZIP) [file pone.0231923.s002.zip › Fig 2 original data/Morphological change/1E8/control/control-2.tif]

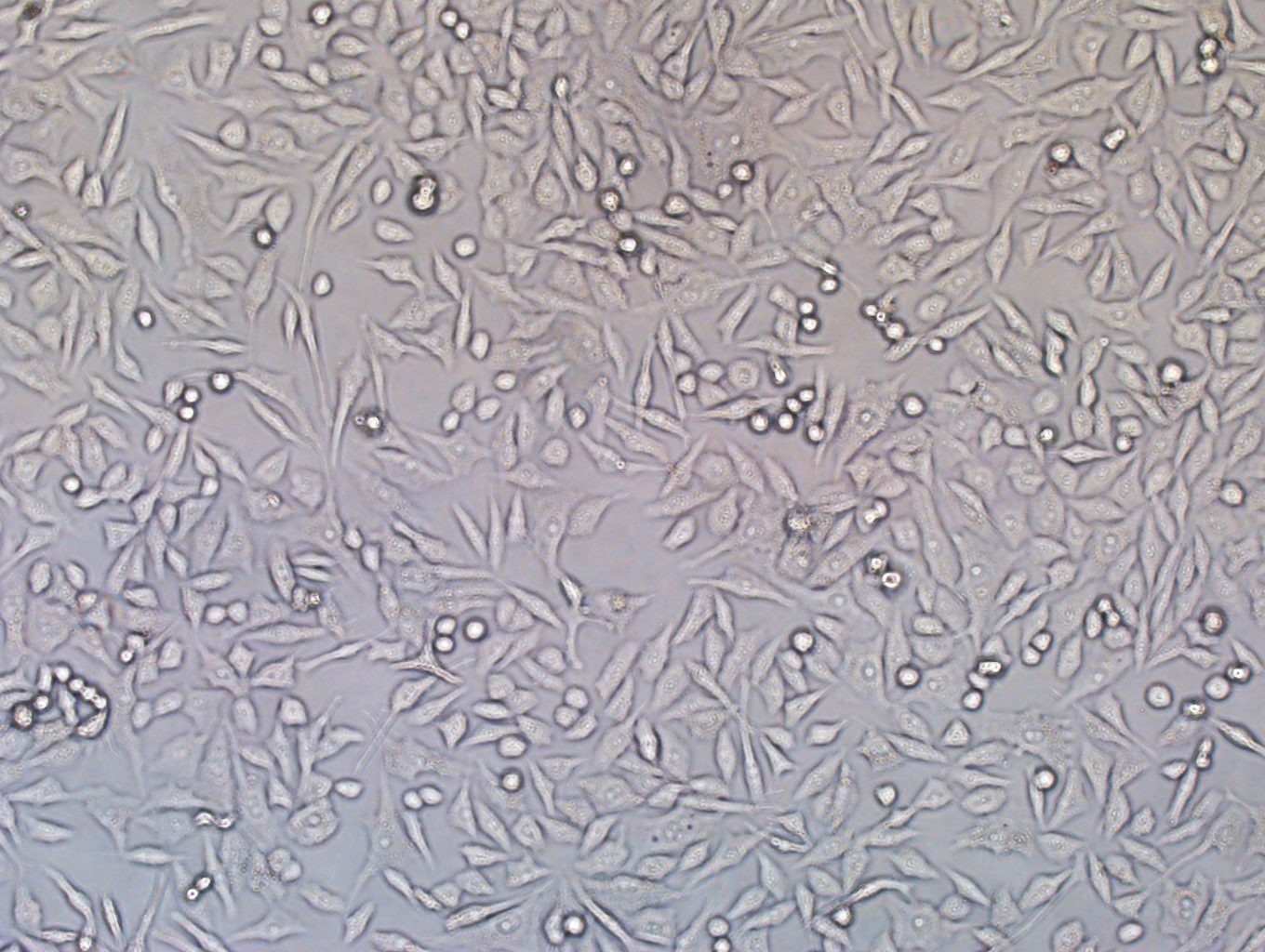

Supplement: S2 File — (ZIP) [file pone.0231923.s002.zip › Fig 2 original data/Morphological change/1E8/control/control-3.tif]

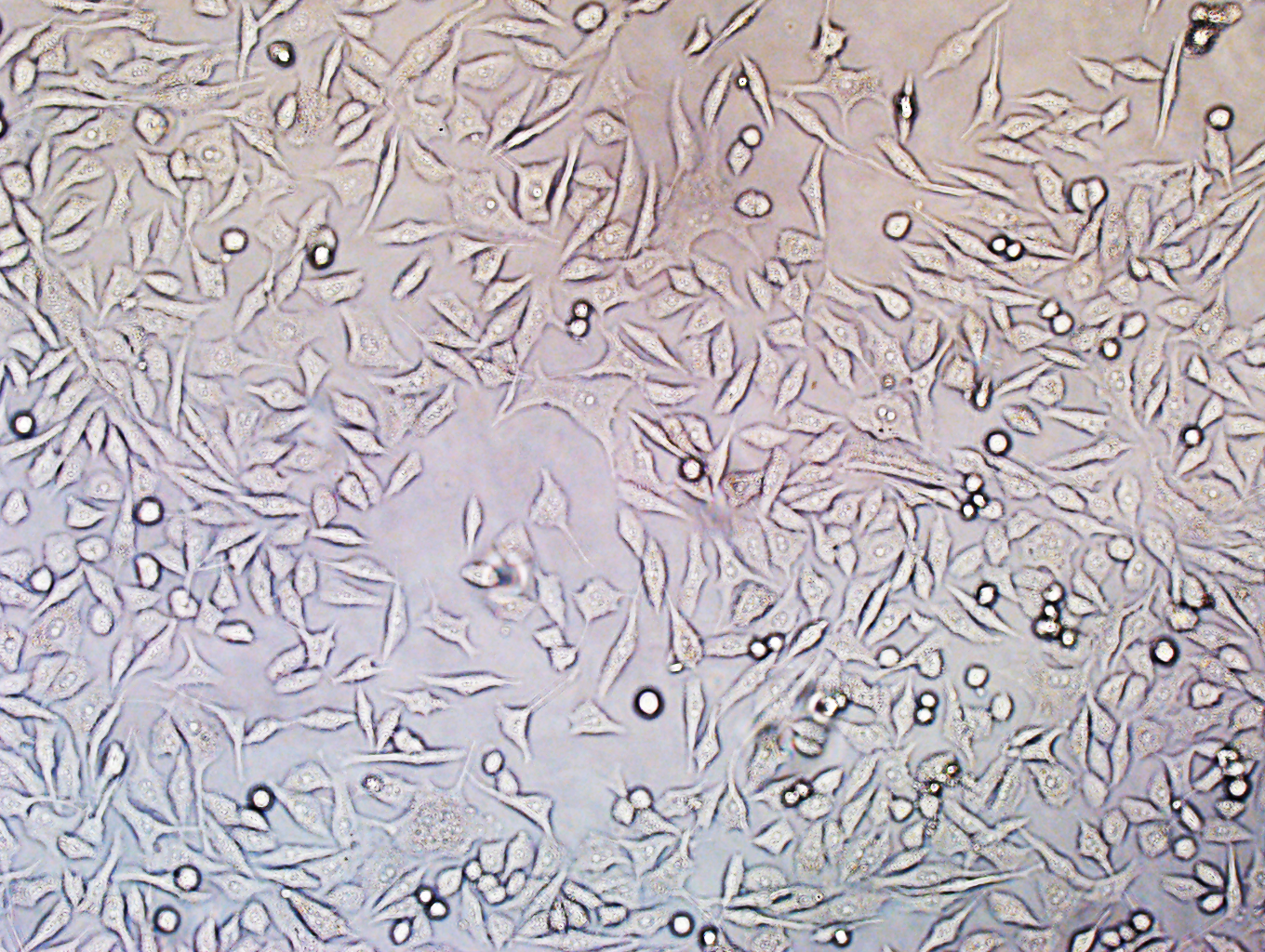

Supplement: S2 File — (ZIP) [file pone.0231923.s002.zip › Fig 2 original data/Morphological change/1E8/sv-TMTP1-DKK/sv-TMTP1-DKK-1.tif]

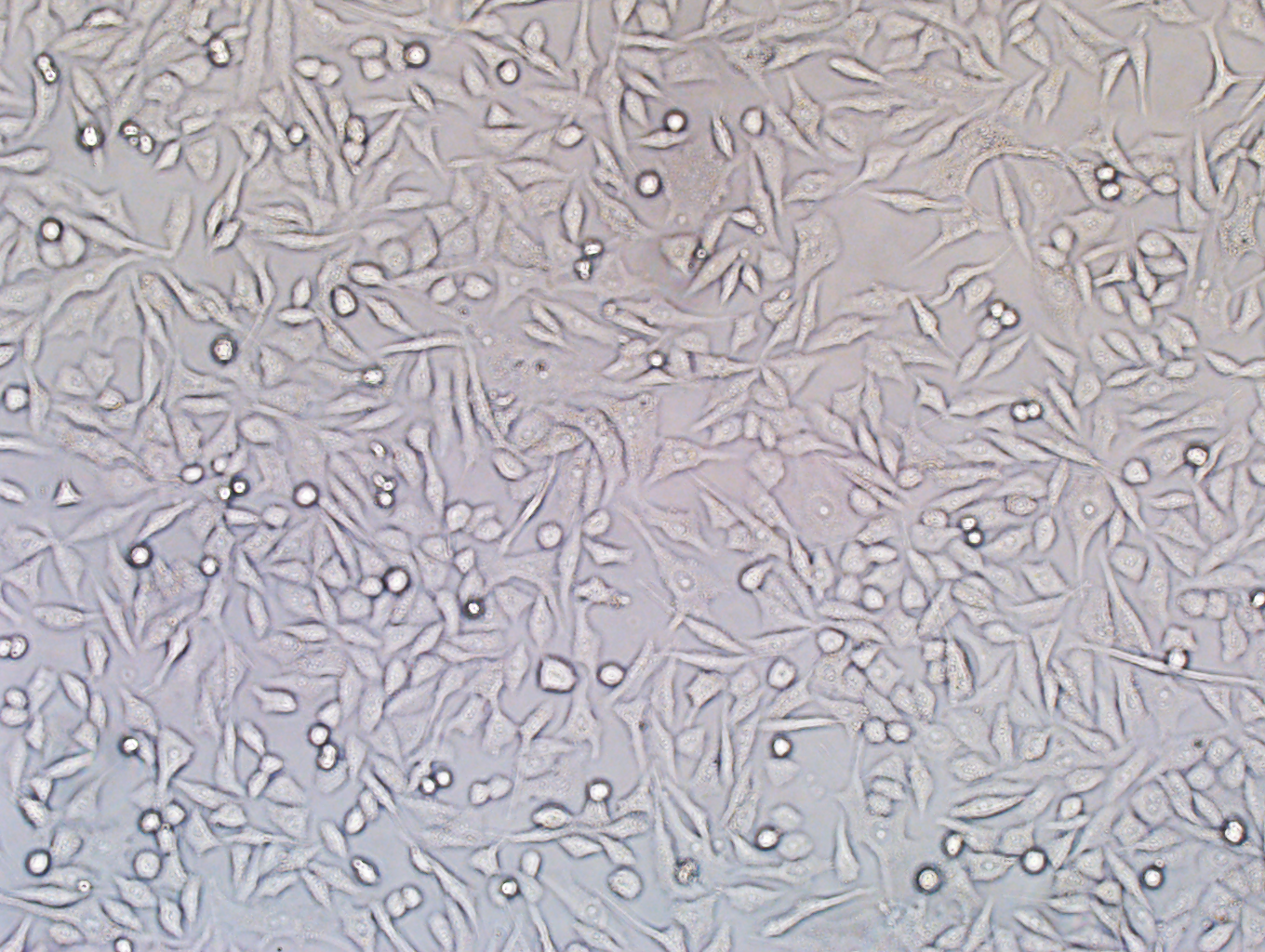

Supplement: S2 File — (ZIP) [file pone.0231923.s002.zip › Fig 2 original data/Morphological change/1E8/sv-TMTP1-DKK/sv-TMTP1-DKK-2.tif]

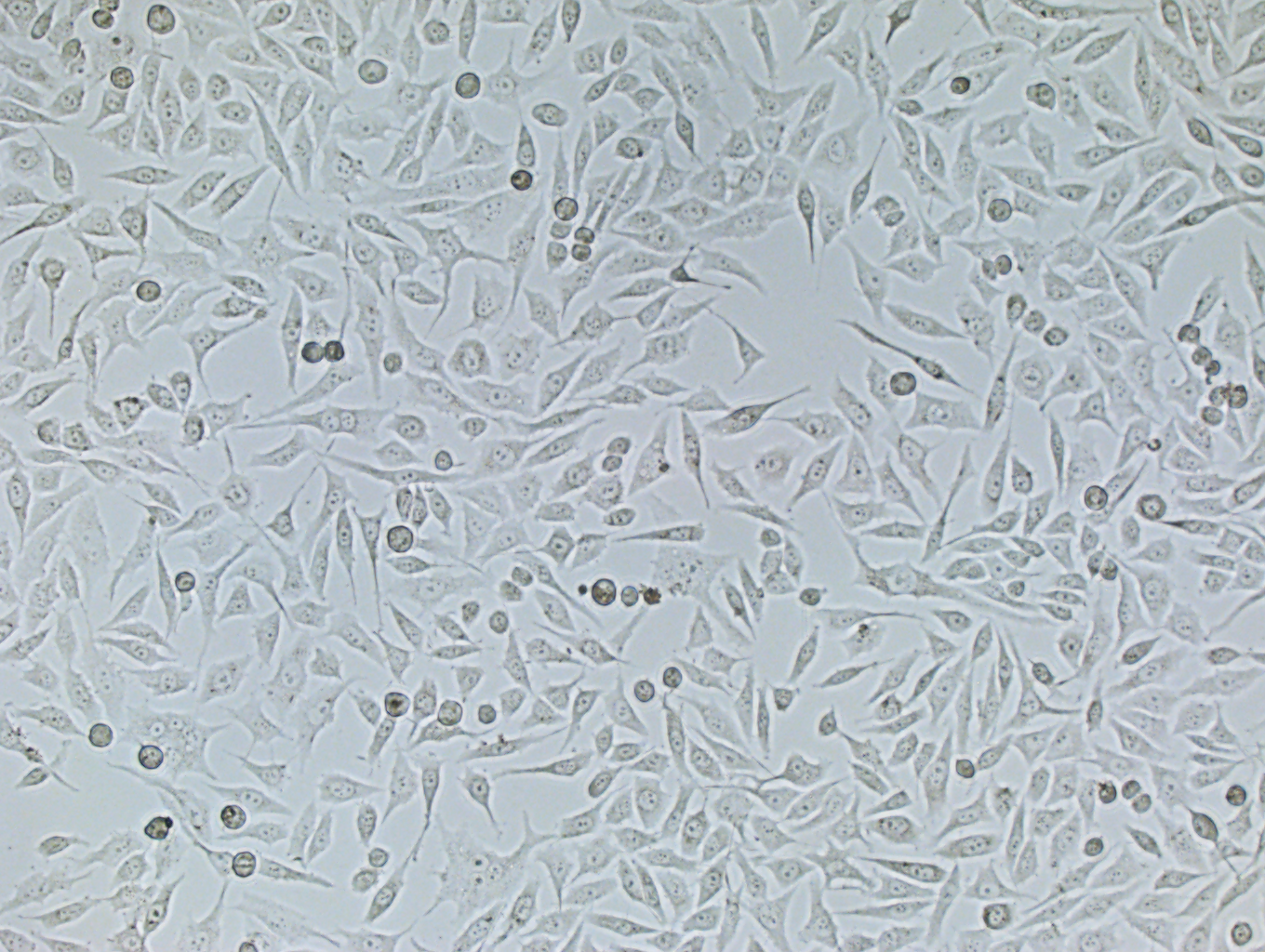

Supplement: S2 File — (ZIP) [file pone.0231923.s002.zip › Fig 2 original data/Morphological change/1E8/sv-TMTP1-DKK/sv-TMTP1-DKK-3.tif]

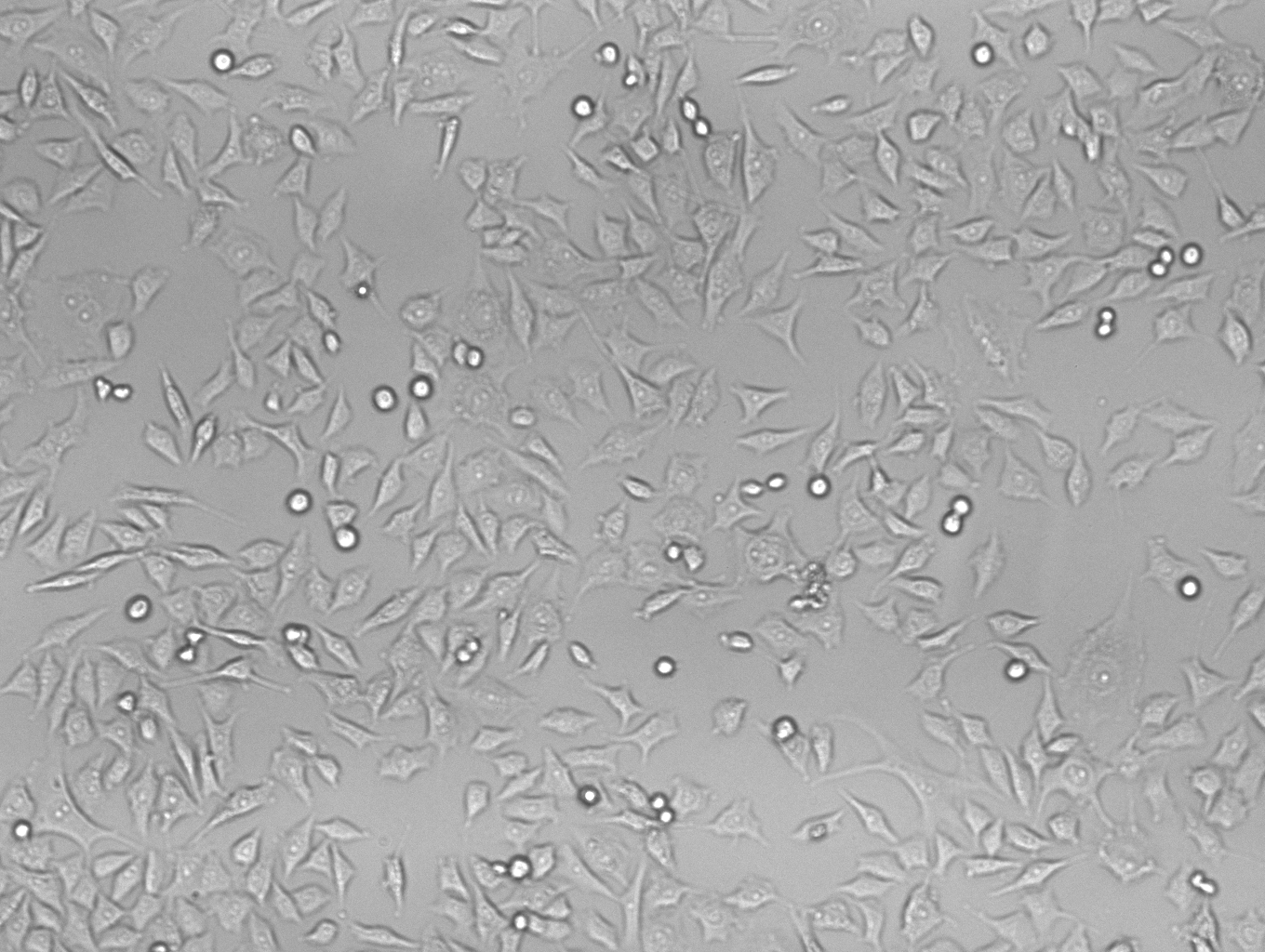

Supplement: S2 File — (ZIP) [file pone.0231923.s002.zip › Fig 2 original data/Morphological change/1E8-2/control/control-1.tif]

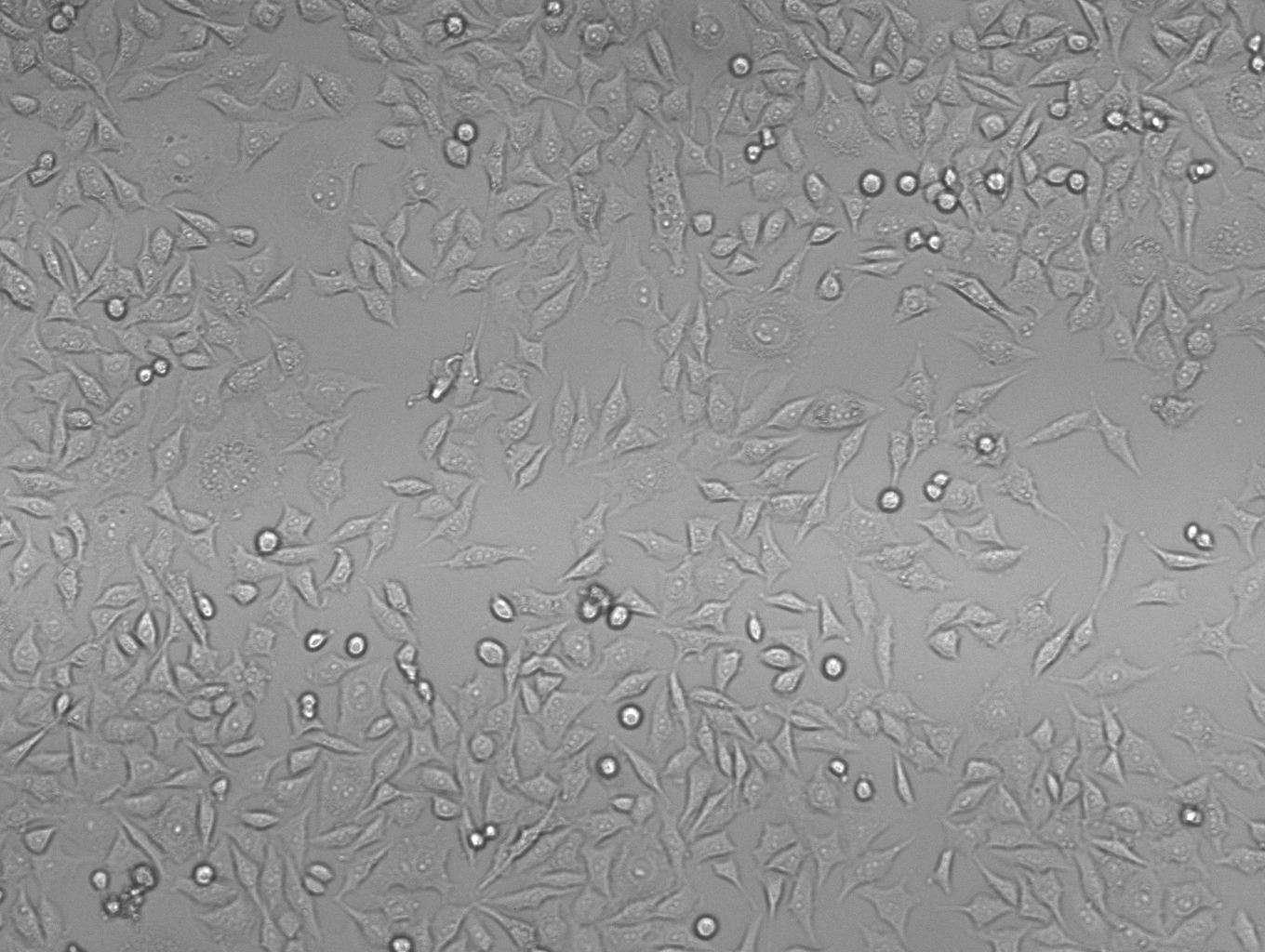

Supplement: S2 File — (ZIP) [file pone.0231923.s002.zip › Fig 2 original data/Morphological change/1E8-2/control/control-2.tif]

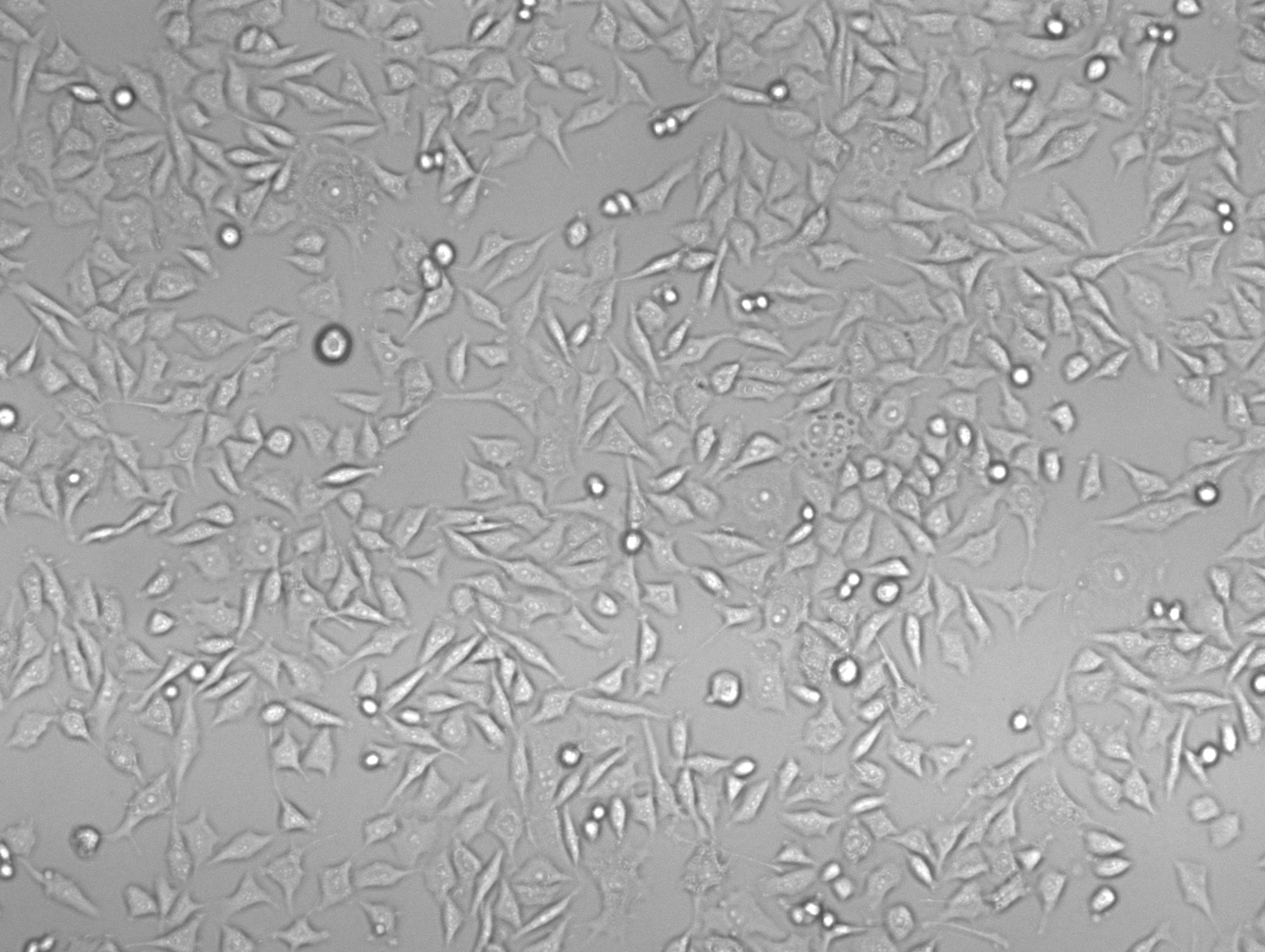

Supplement: S2 File — (ZIP) [file pone.0231923.s002.zip › Fig 2 original data/Morphological change/1E8-2/control/control-3.tif]

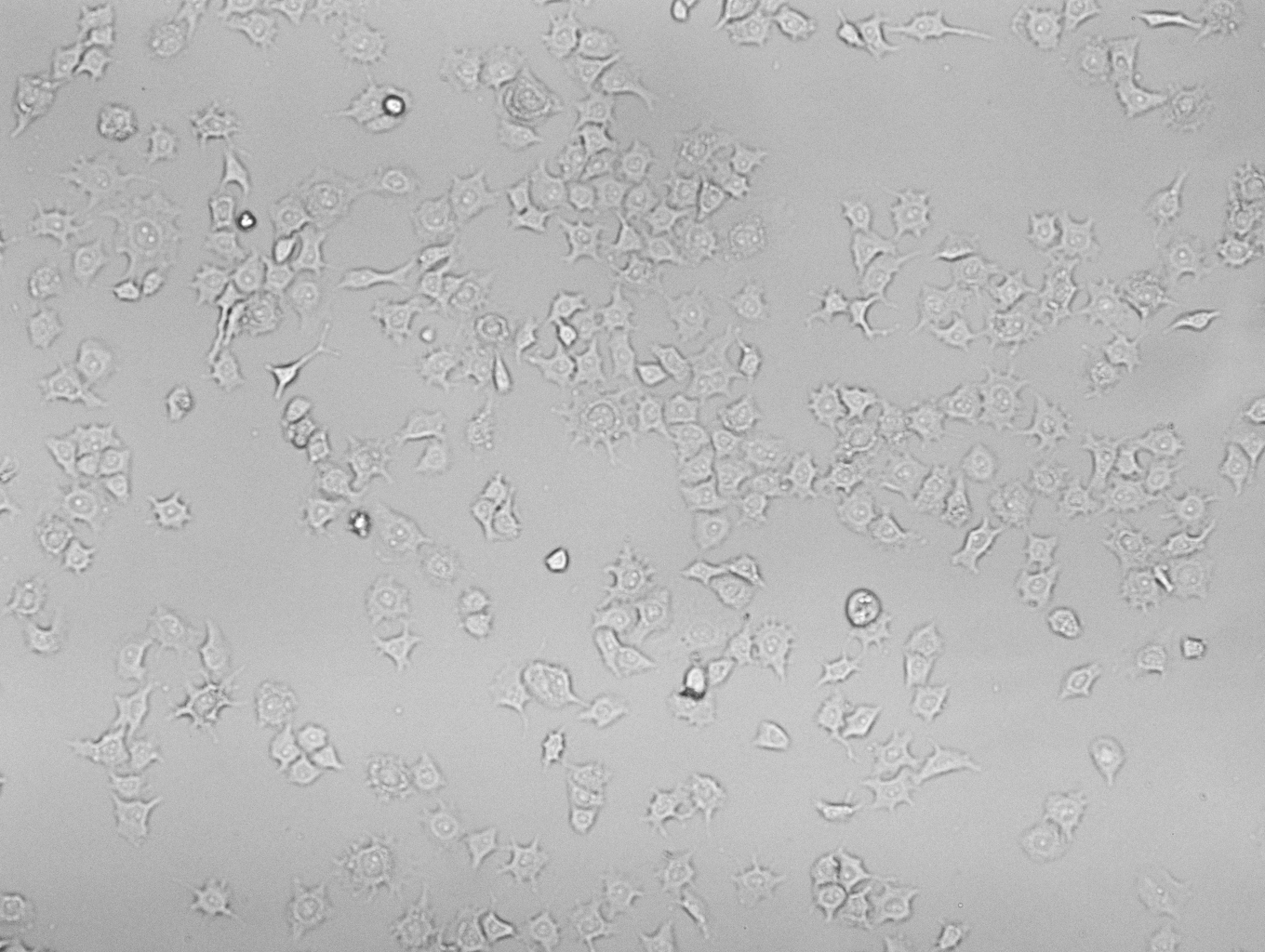

Supplement: S2 File — (ZIP) [file pone.0231923.s002.zip › Fig 2 original data/Morphological change/1E8-2/TMTP1-DKK/TMTP1-DKK-1.tif]

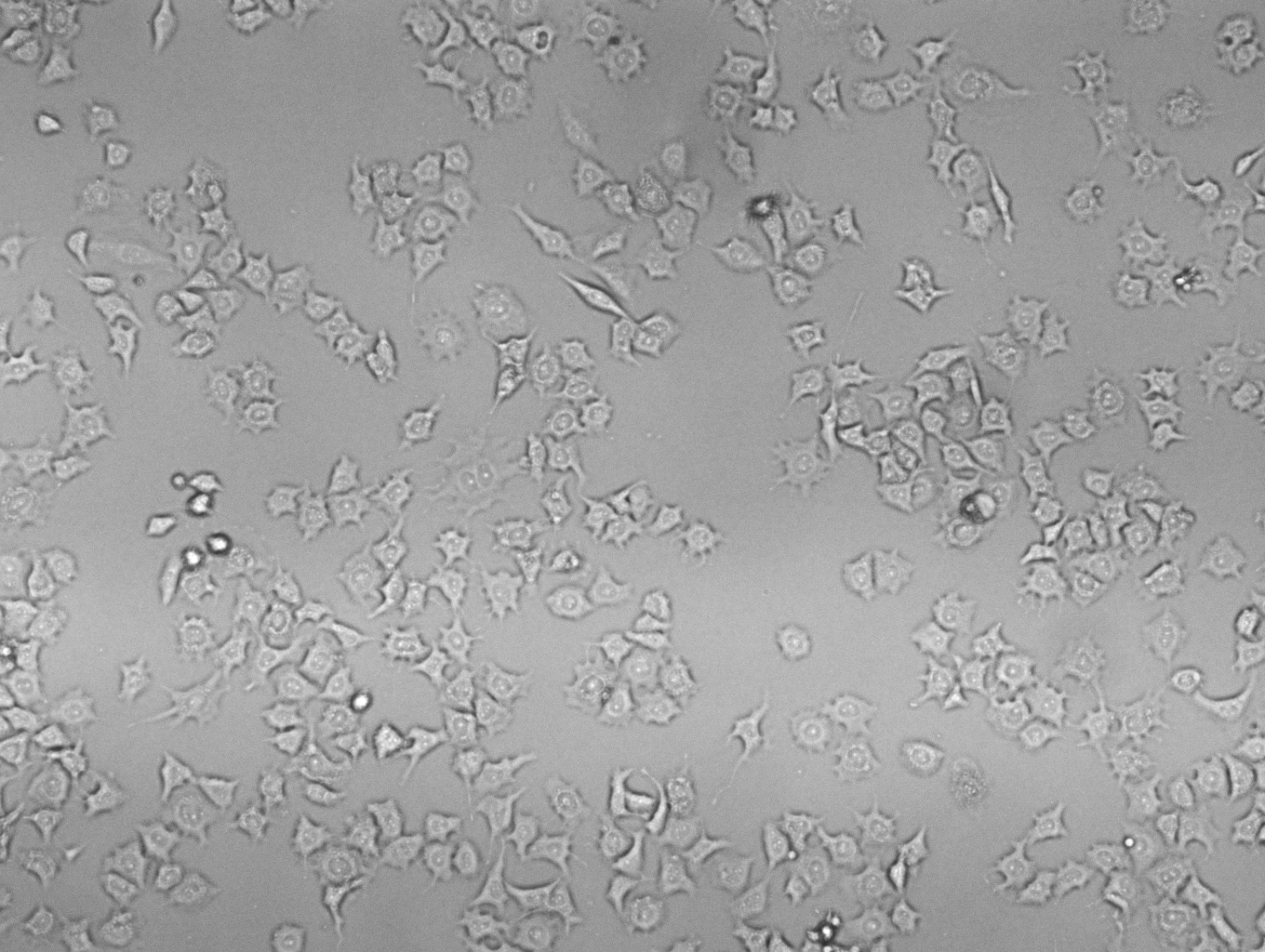

Supplement: S2 File — (ZIP) [file pone.0231923.s002.zip › Fig 2 original data/Morphological change/1E8-2/TMTP1-DKK/TMTP1-DKK-2.tif]

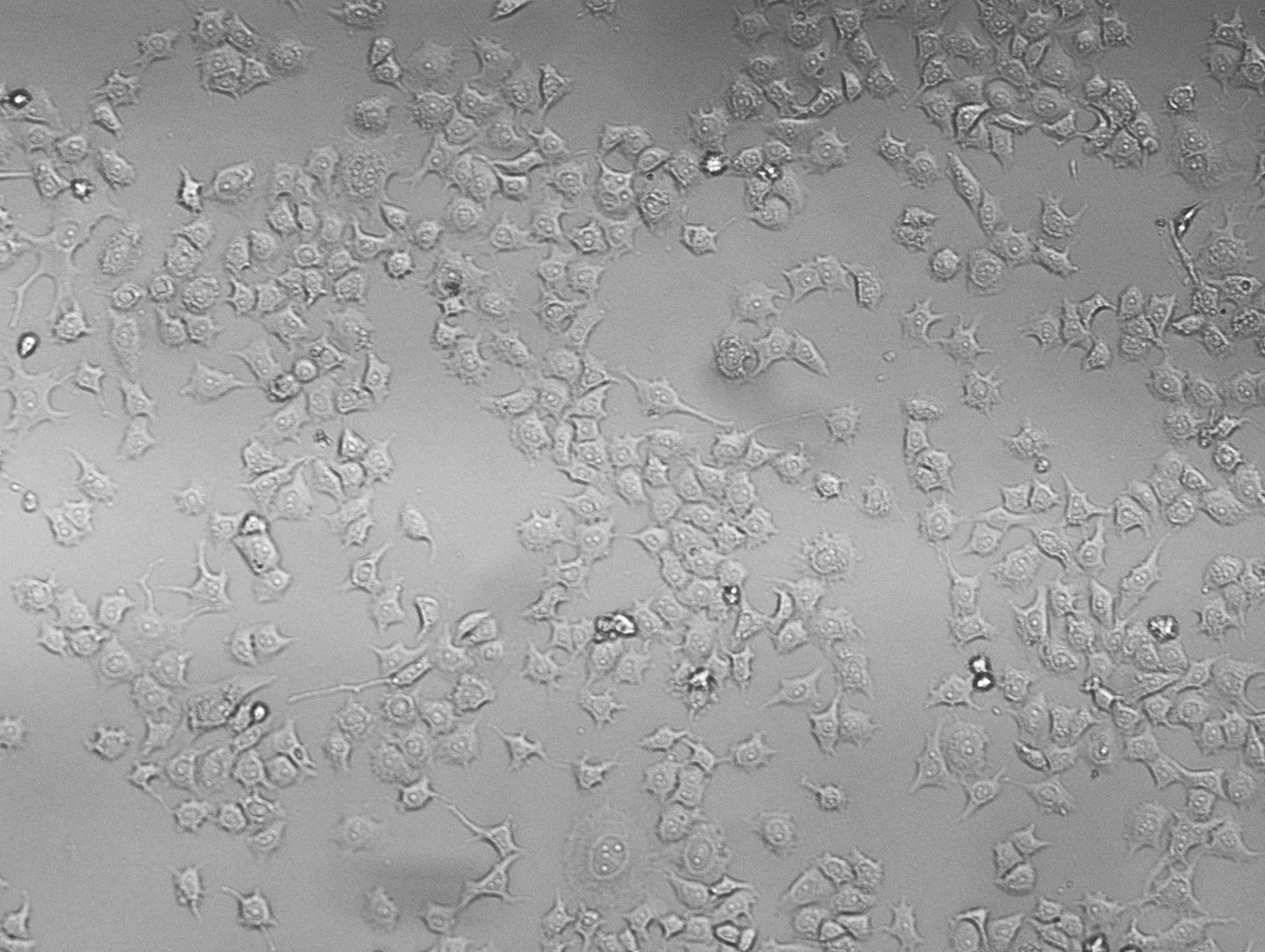

Supplement: S2 File — (ZIP) [file pone.0231923.s002.zip › Fig 2 original data/Morphological change/1E8-2/TMTP1-DKK/TMTP1-DKK-3.tif]

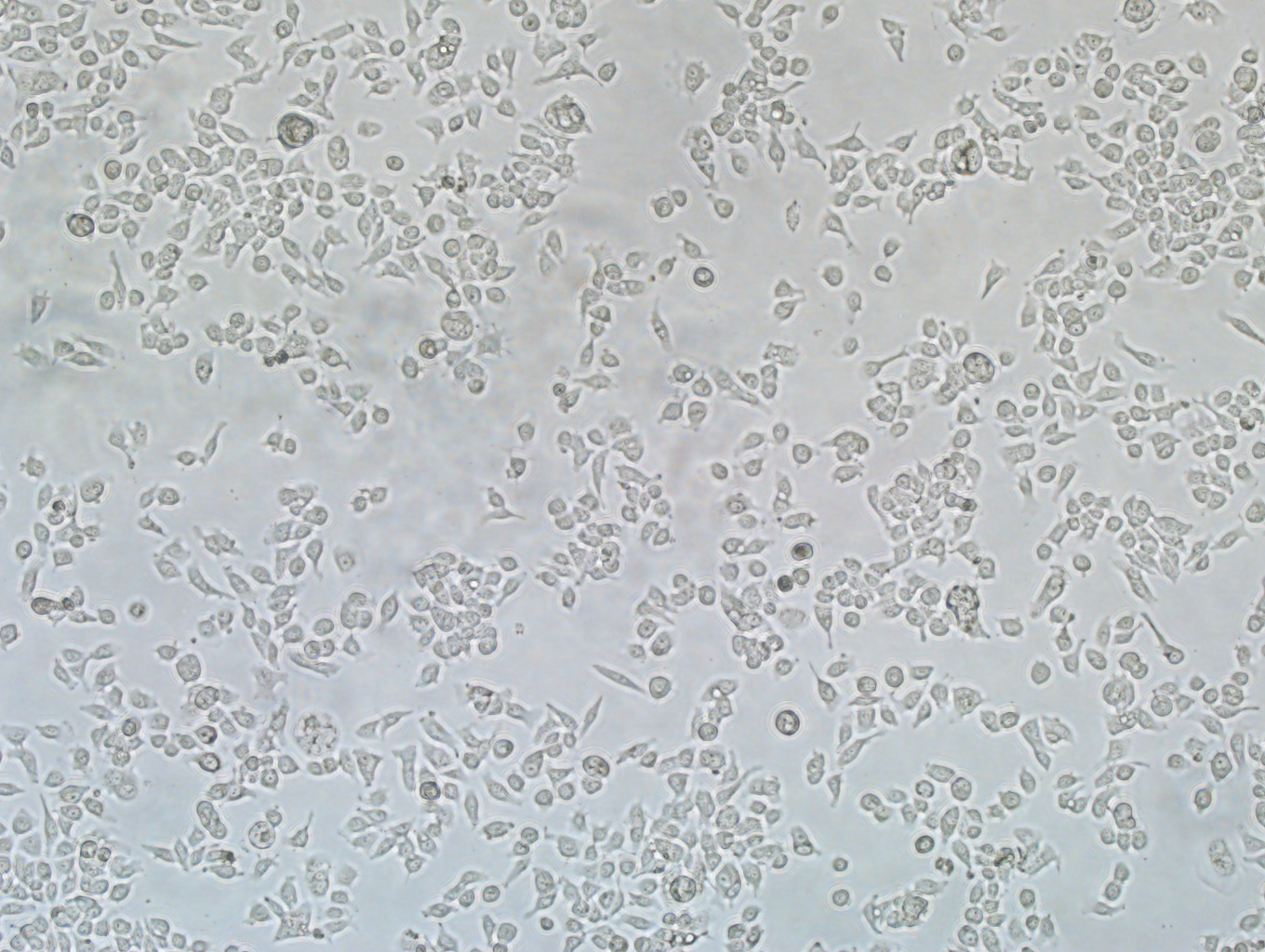

Supplement: S2 File — (ZIP) [file pone.0231923.s002.zip › Fig 2 original data/Morphological change/MKN45/control/control-1.tif]

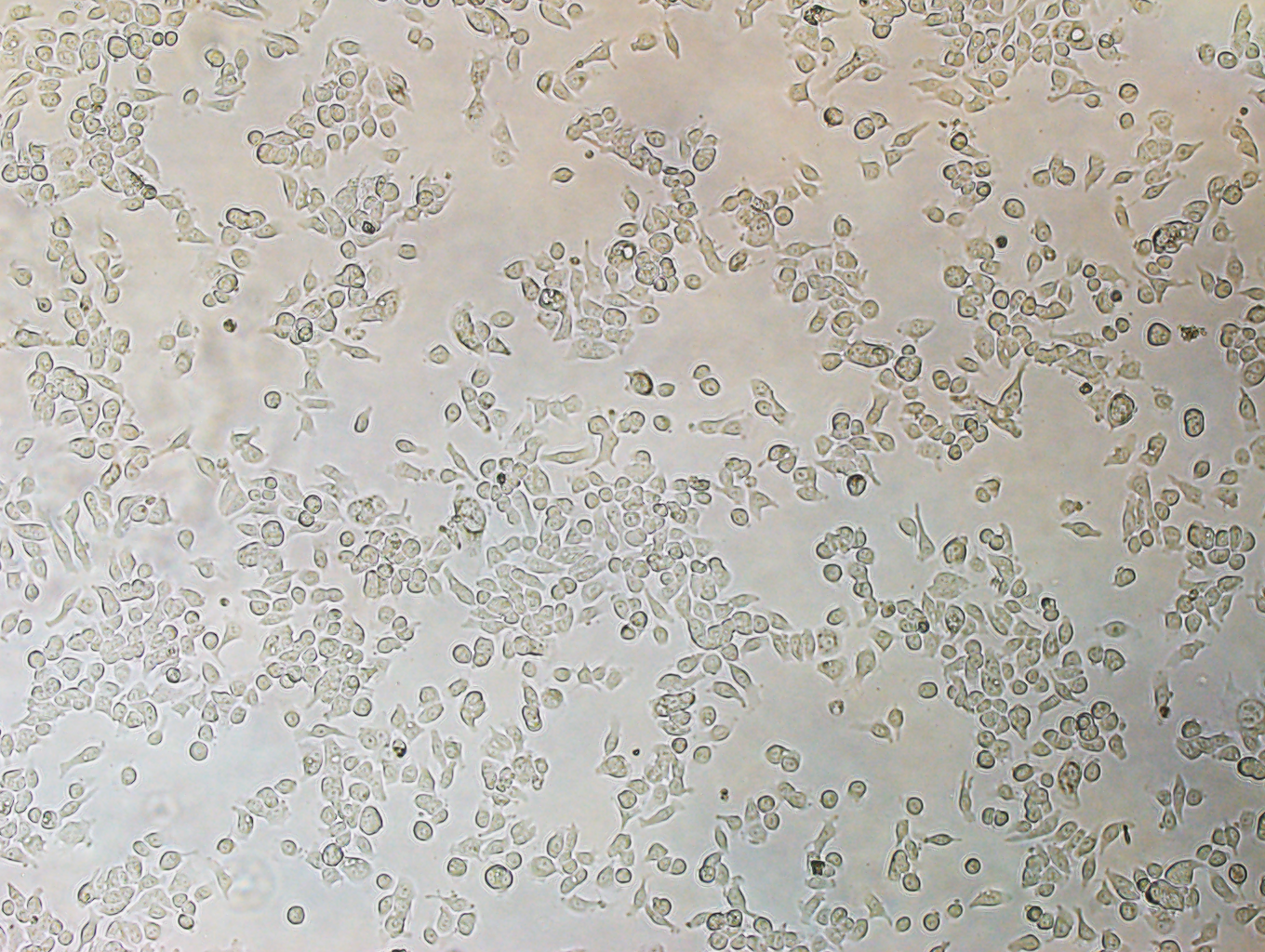

Supplement: S2 File — (ZIP) [file pone.0231923.s002.zip › Fig 2 original data/Morphological change/MKN45/control/control-2.tif]

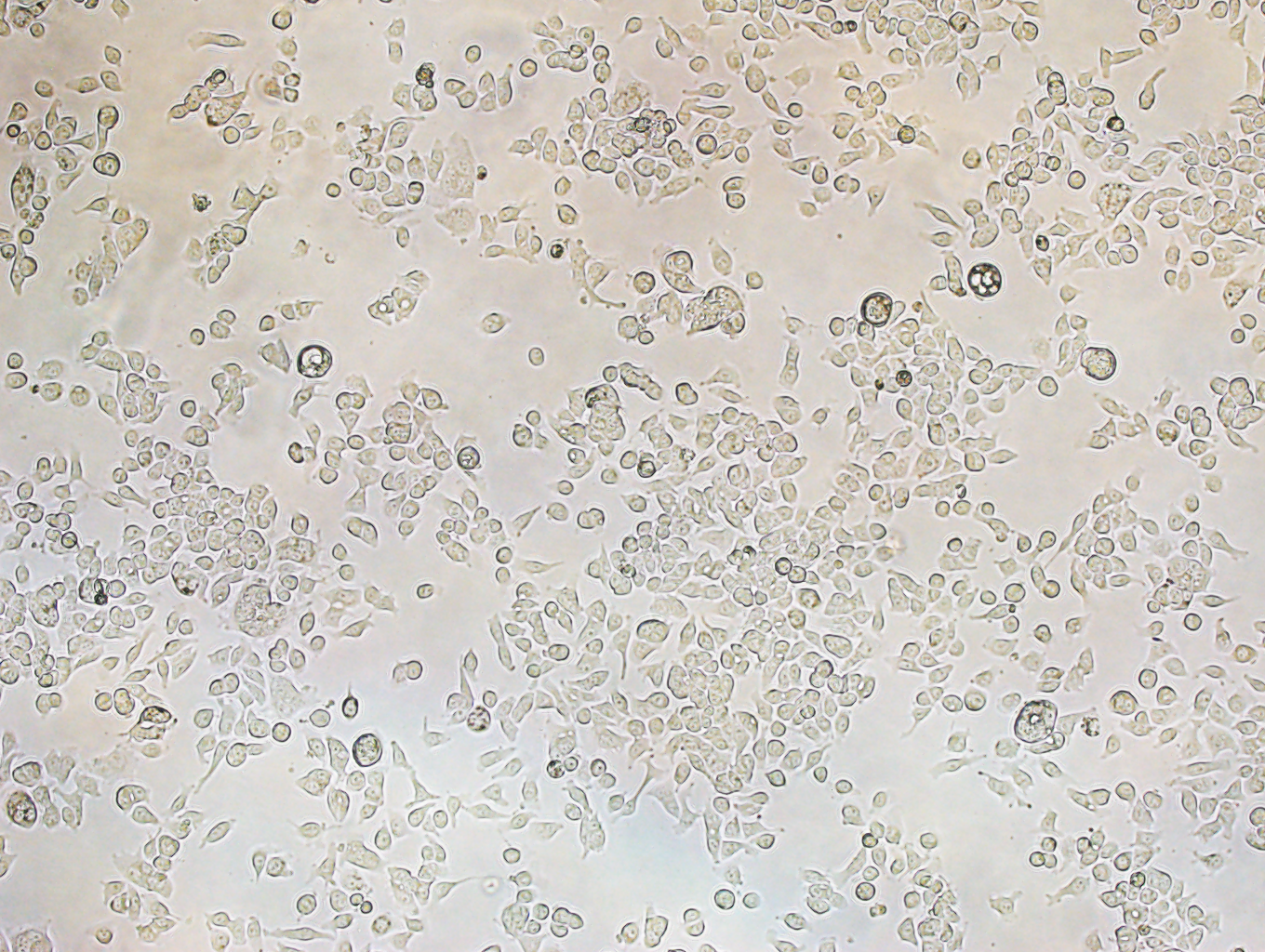

Supplement: S2 File — (ZIP) [file pone.0231923.s002.zip › Fig 2 original data/Morphological change/MKN45/control/control-3.tif]

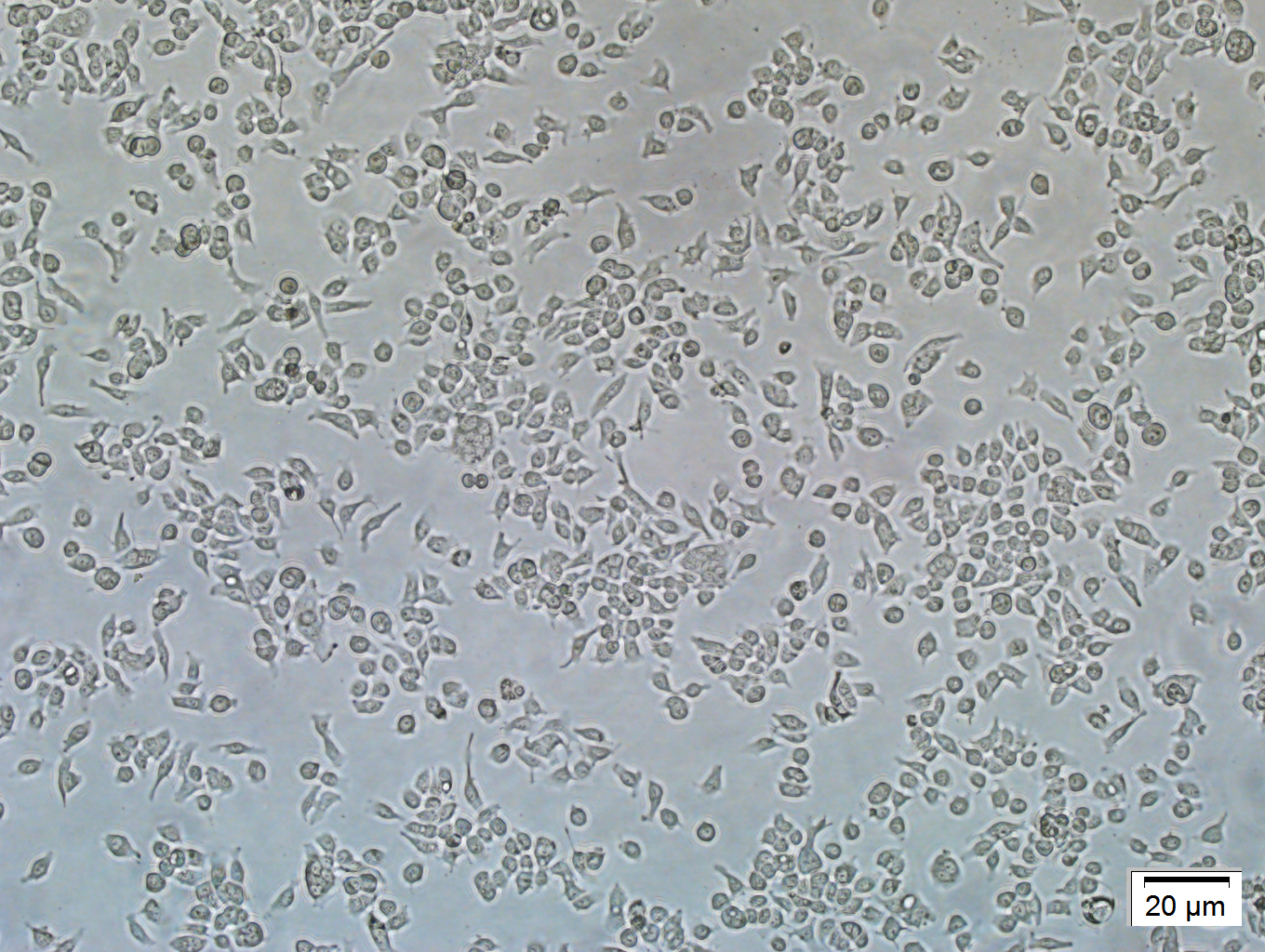

Supplement: S2 File — (ZIP) [file pone.0231923.s002.zip › Fig 2 original data/Morphological change/MKN45/sv-TMTP1-DKK/sv-TMTP1-DKK-1.tif]

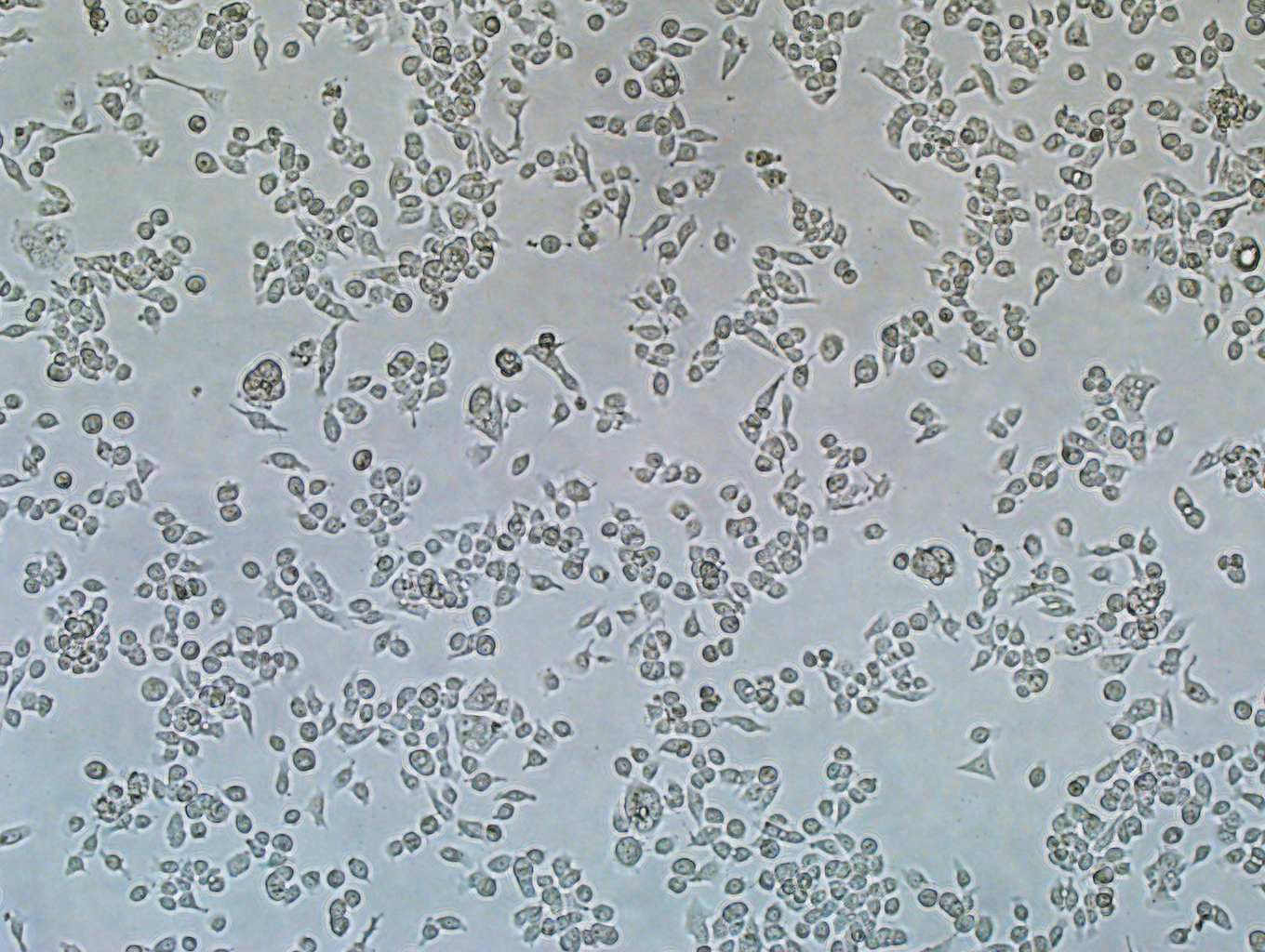

Supplement: S2 File — (ZIP) [file pone.0231923.s002.zip › Fig 2 original data/Morphological change/MKN45/sv-TMTP1-DKK/sv-TMTP1-DKK-2.tif]

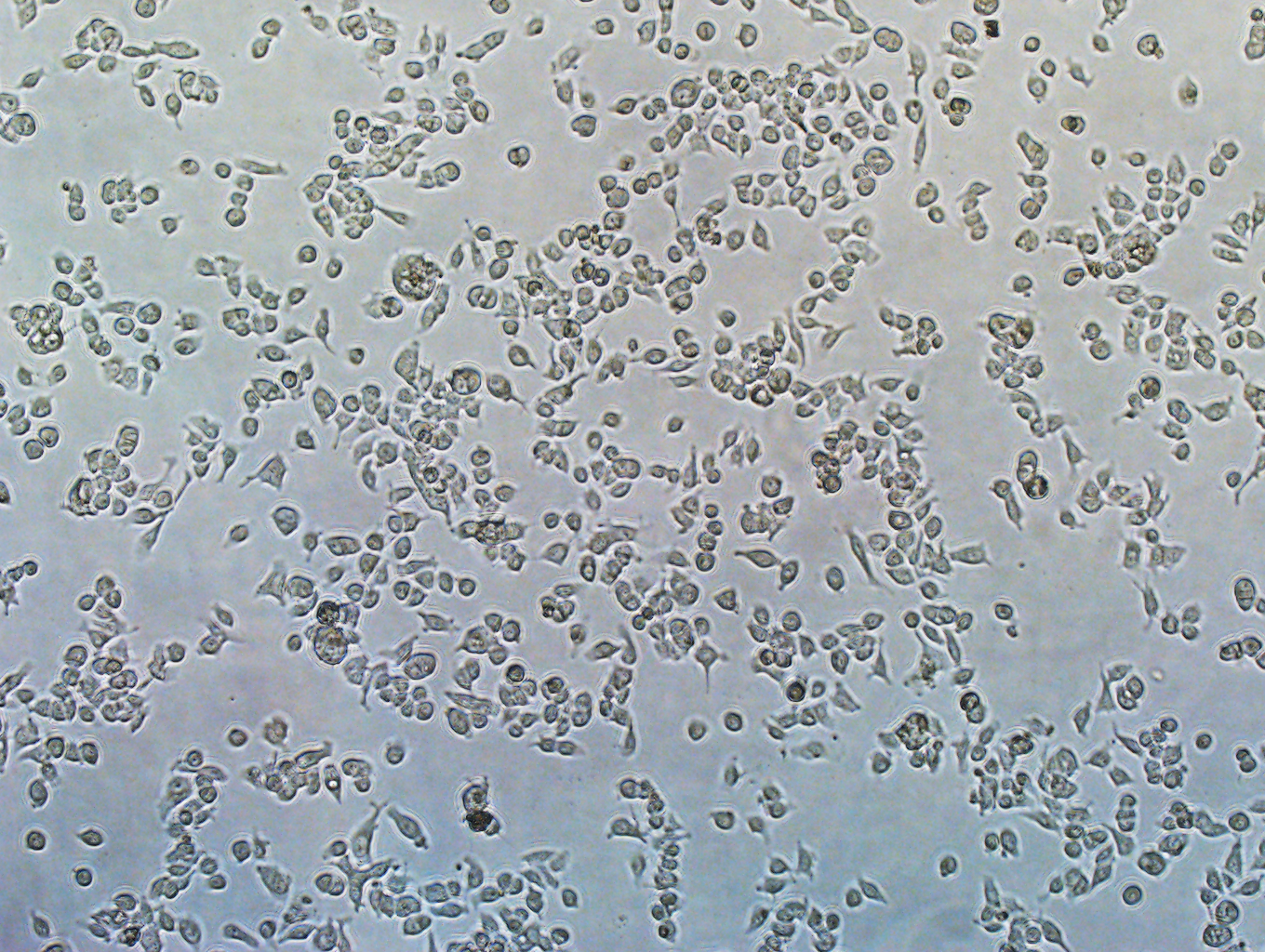

Supplement: S2 File — (ZIP) [file pone.0231923.s002.zip › Fig 2 original data/Morphological change/MKN45/sv-TMTP1-DKK/sv-TMTP1-DKK-3.tif]

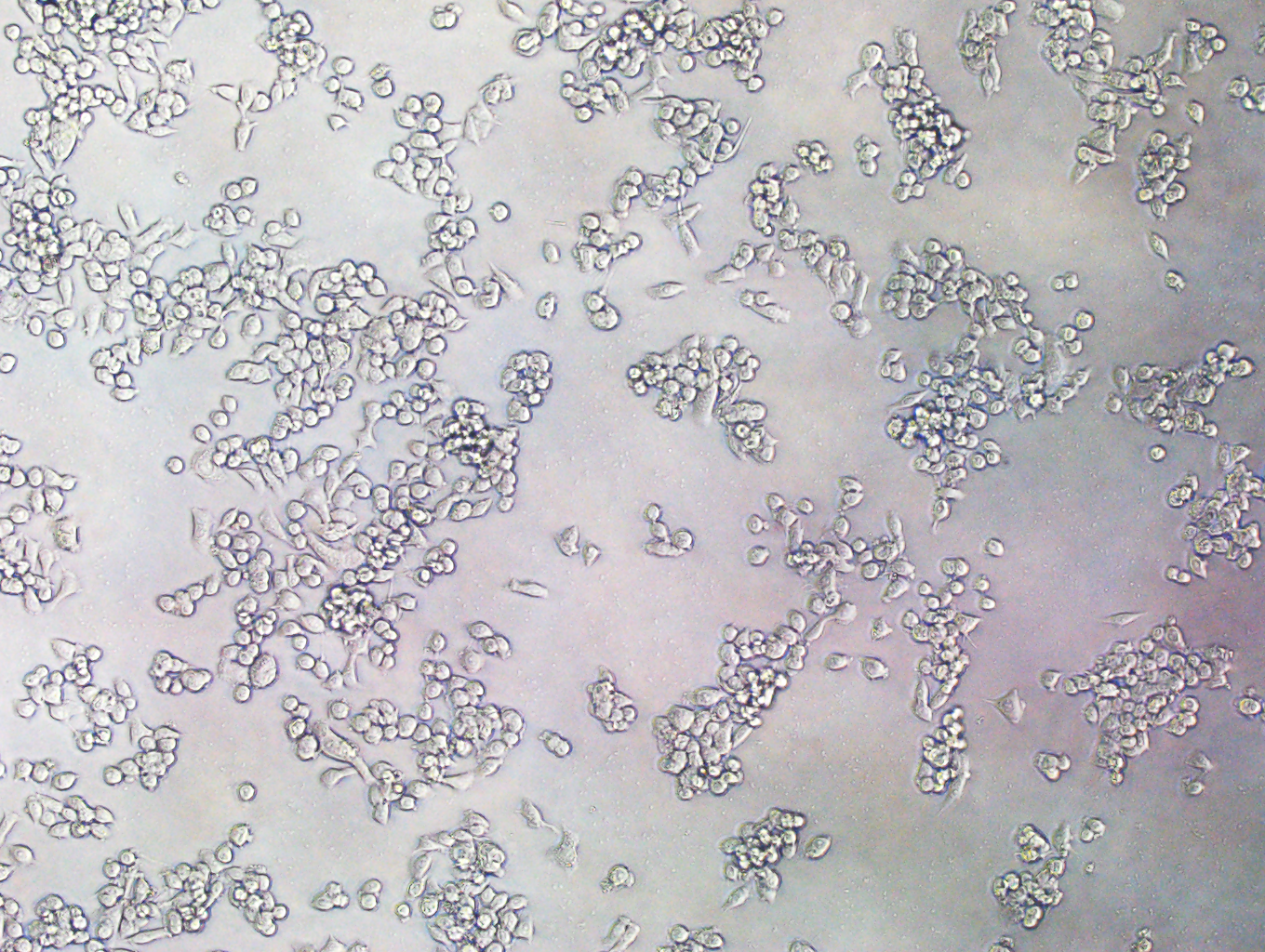

Supplement: S2 File — (ZIP) [file pone.0231923.s002.zip › Fig 2 original data/Morphological change/MKN45-2/control/control-1.tif]

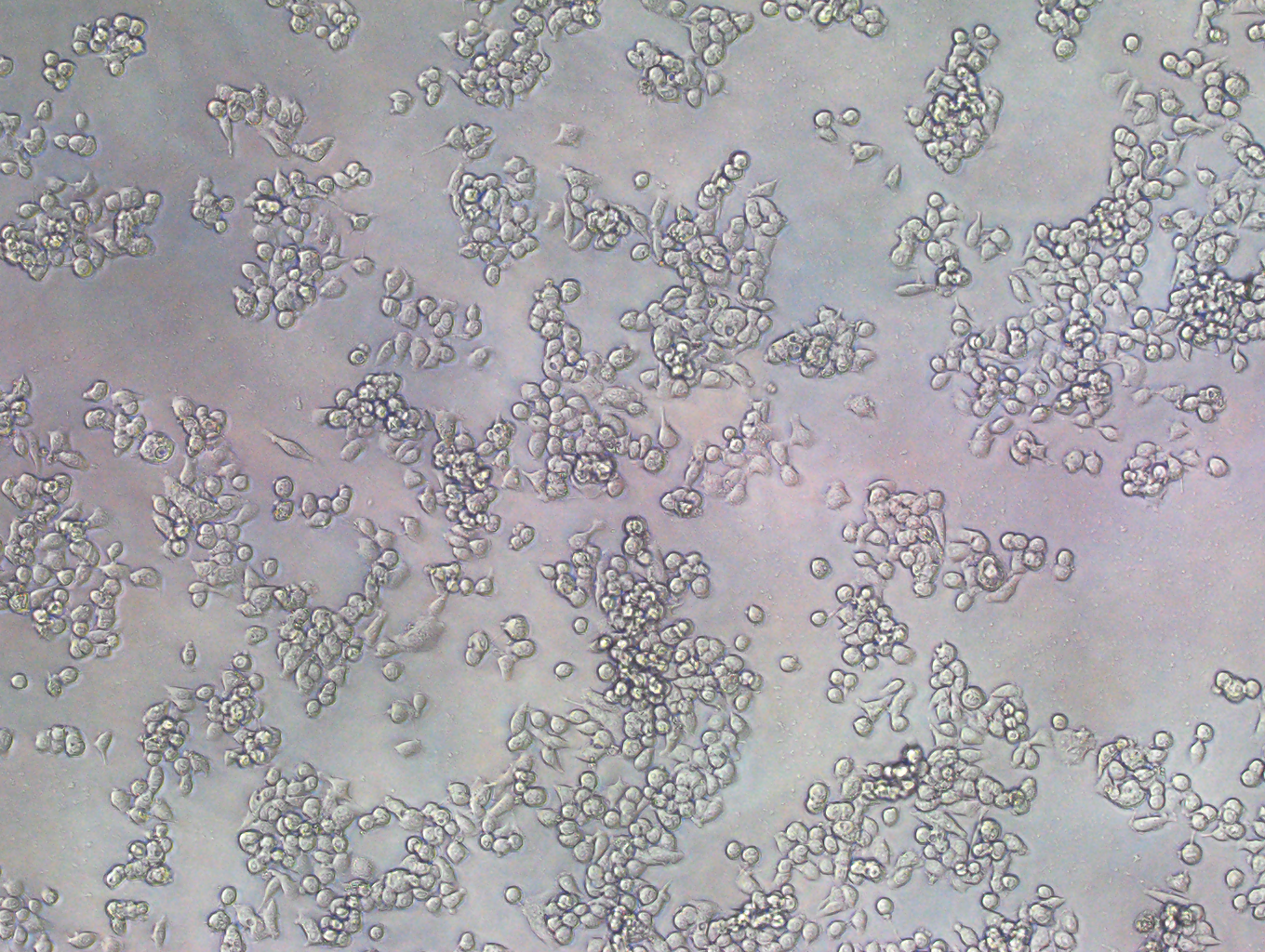

Supplement: S2 File — (ZIP) [file pone.0231923.s002.zip › Fig 2 original data/Morphological change/MKN45-2/control/control-2.tif]

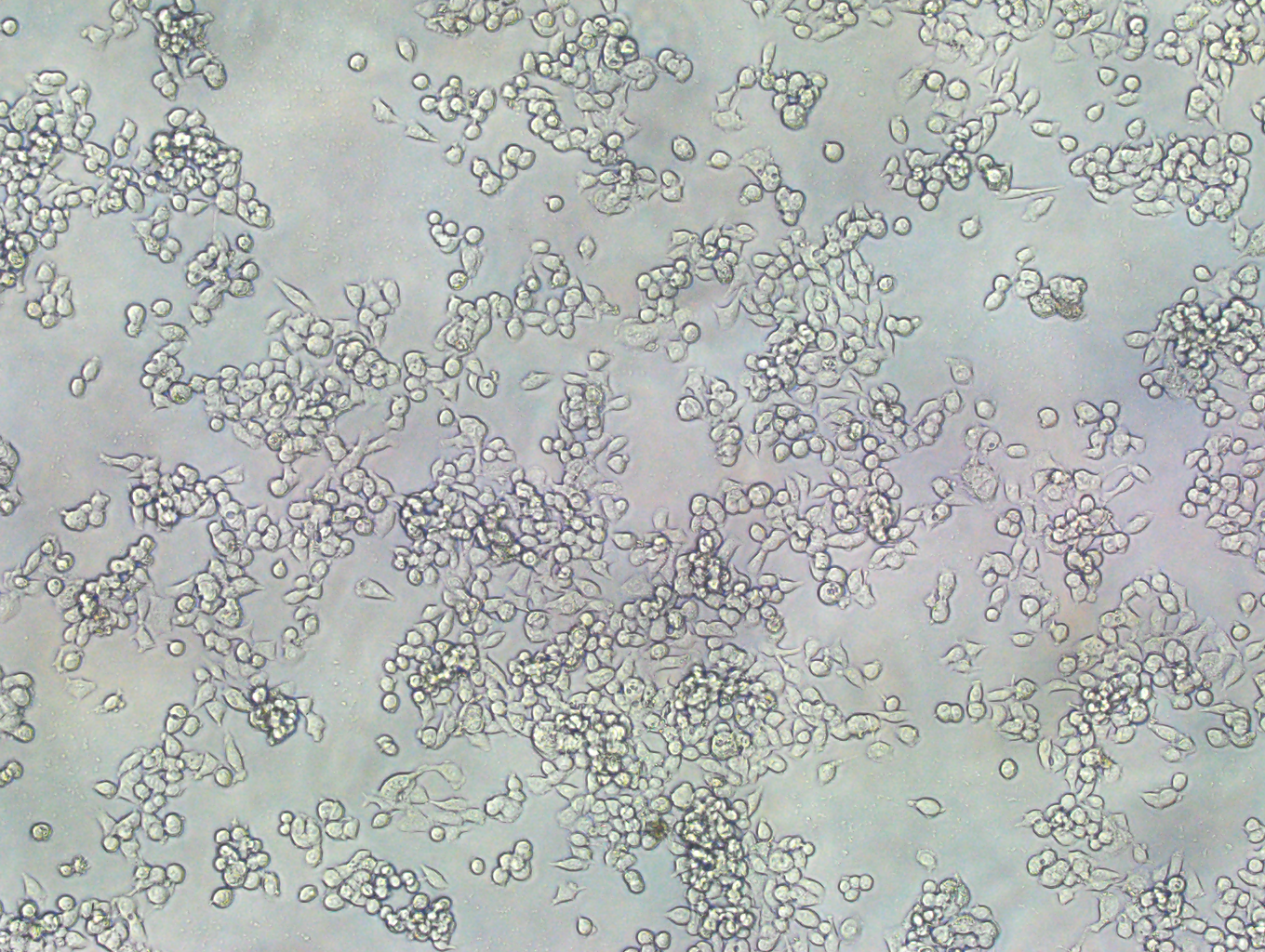

Supplement: S2 File — (ZIP) [file pone.0231923.s002.zip › Fig 2 original data/Morphological change/MKN45-2/control/control-3.tif]

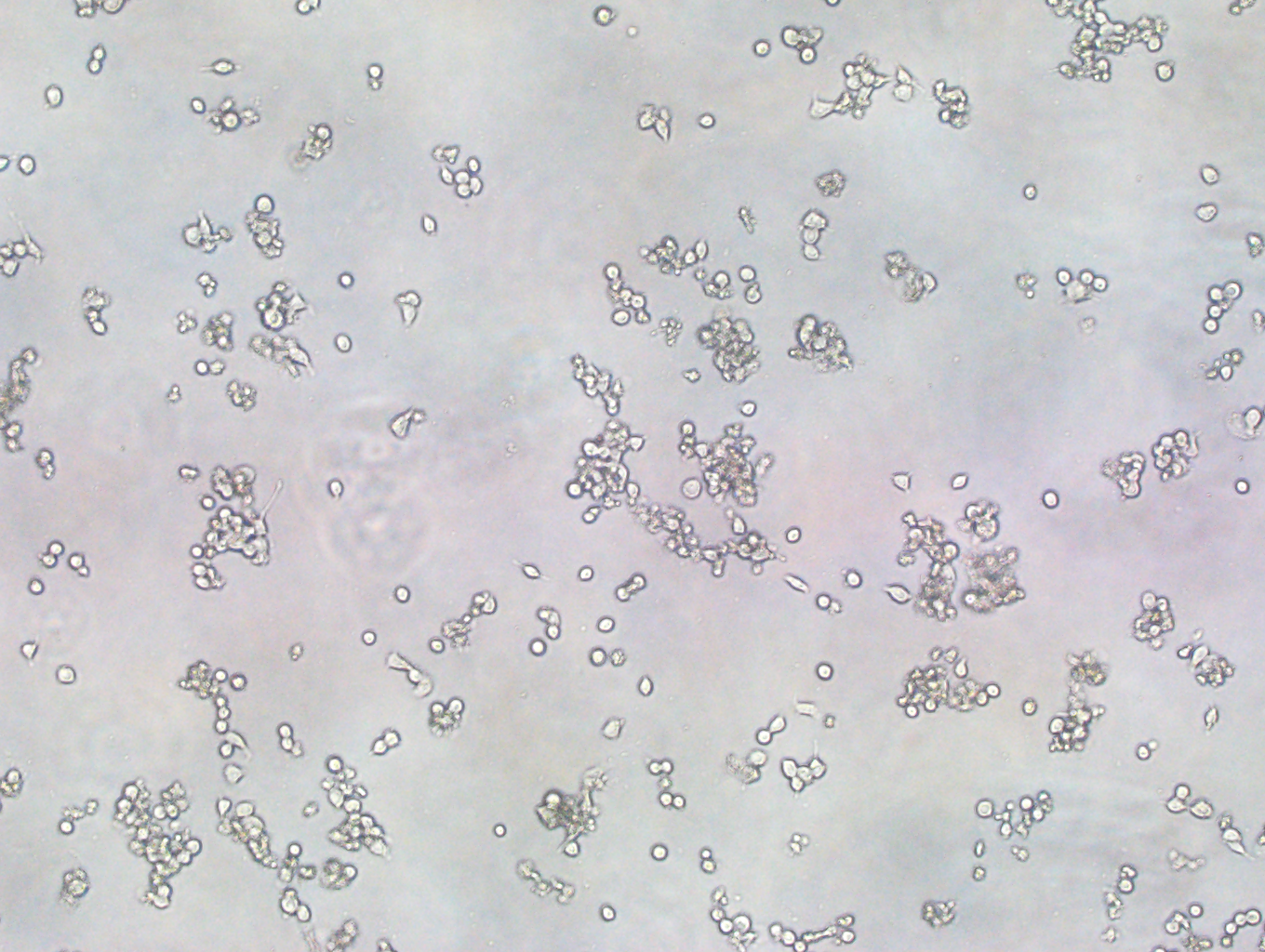

Supplement: S2 File — (ZIP) [file pone.0231923.s002.zip › Fig 2 original data/Morphological change/MKN45-2/TMTP1-DKK/TMTP1-DKK-1.tif]

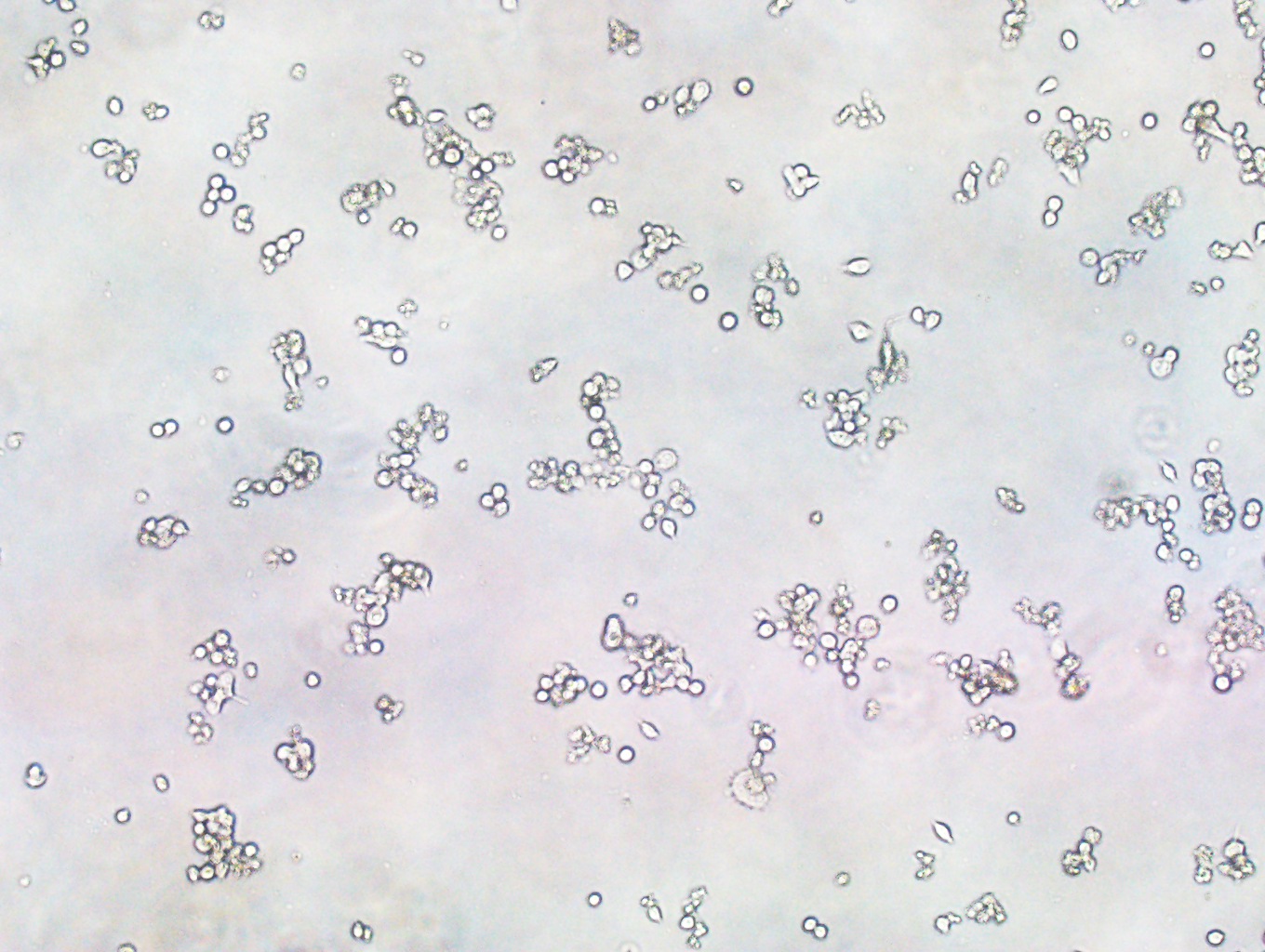

Supplement: S2 File — (ZIP) [file pone.0231923.s002.zip › Fig 2 original data/Morphological change/MKN45-2/TMTP1-DKK/TMTP1-DKK-2.tif]

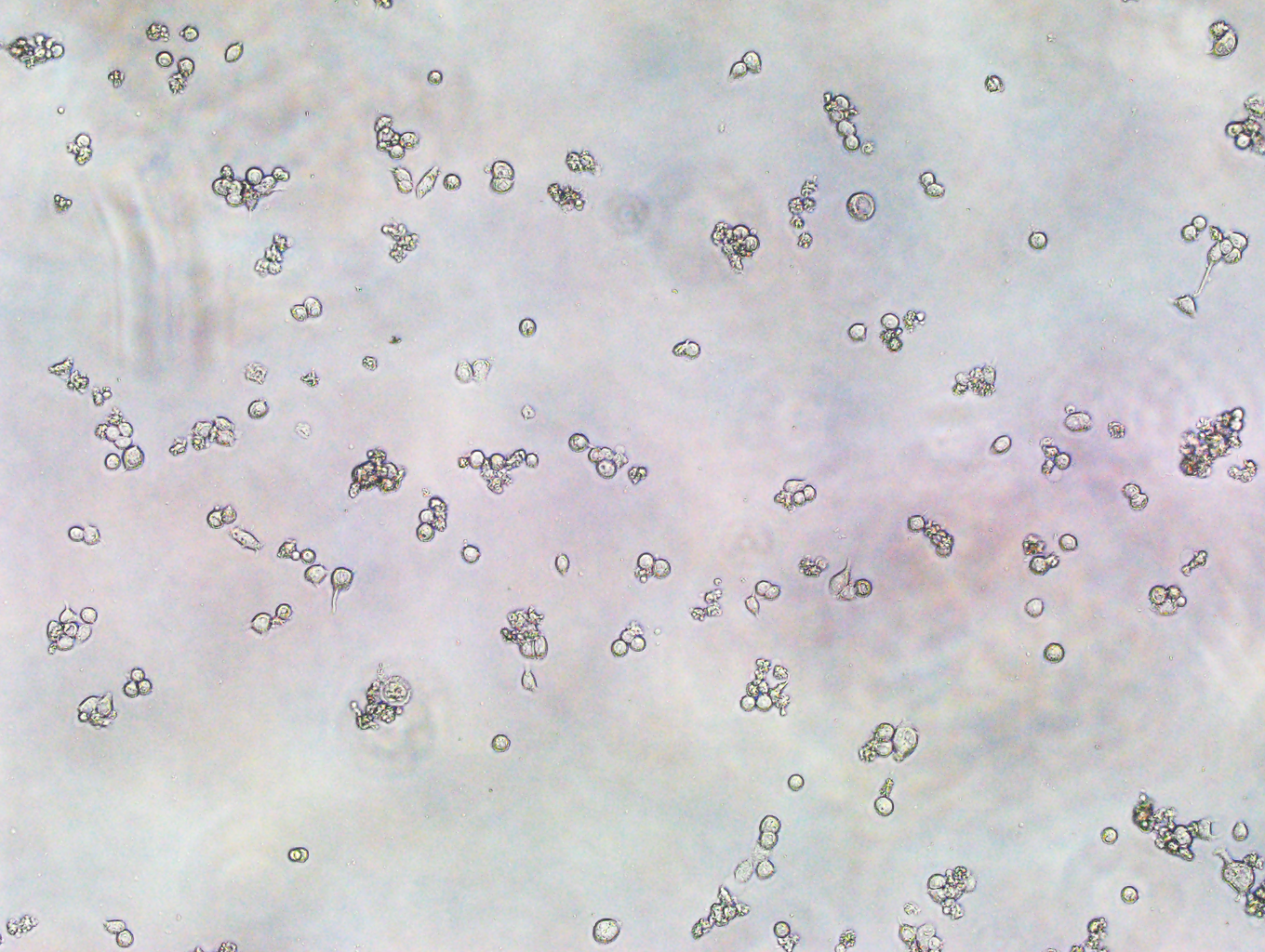

Supplement: S2 File — (ZIP) [file pone.0231923.s002.zip › Fig 2 original data/Morphological change/MKN45-2/TMTP1-DKK/TMTP1-DKK-3.tif]

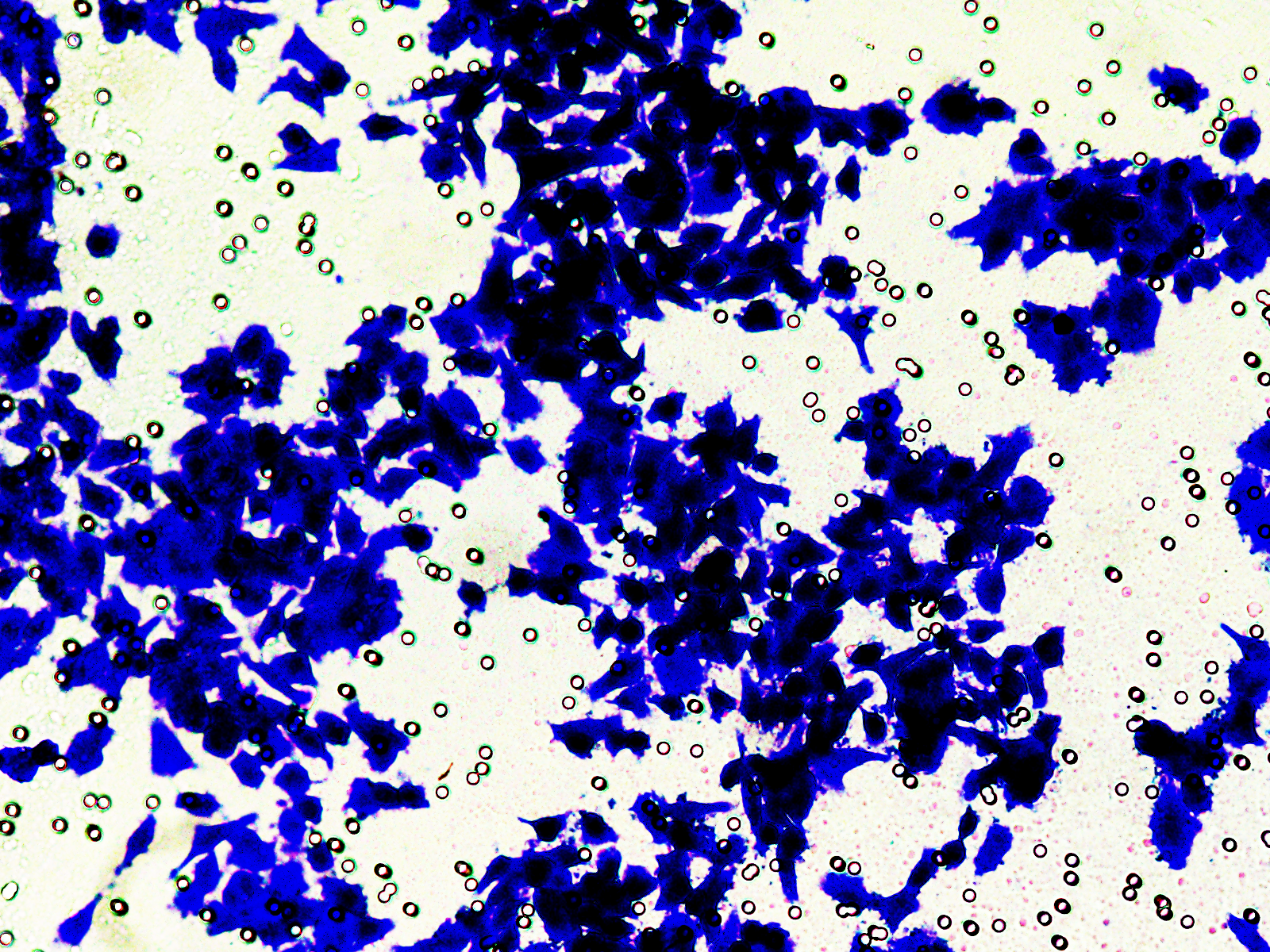

Supplement: S3 File — (ZIP) [file pone.0231923.s003.zip › Transwell/MKN45/Control/control-1.tif]

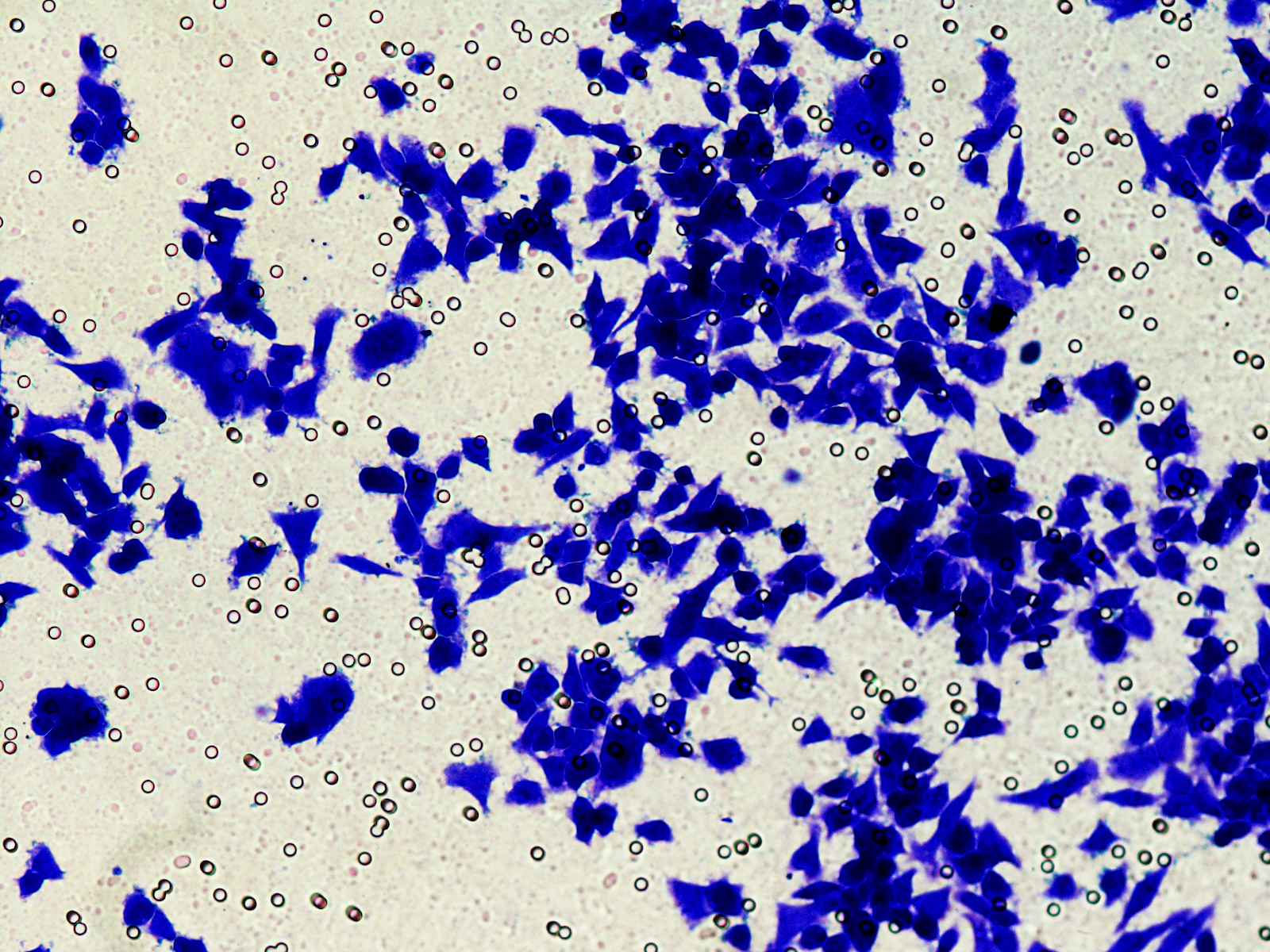

Supplement: S3 File — (ZIP) [file pone.0231923.s003.zip › Transwell/MKN45/Control/control-2.tif]

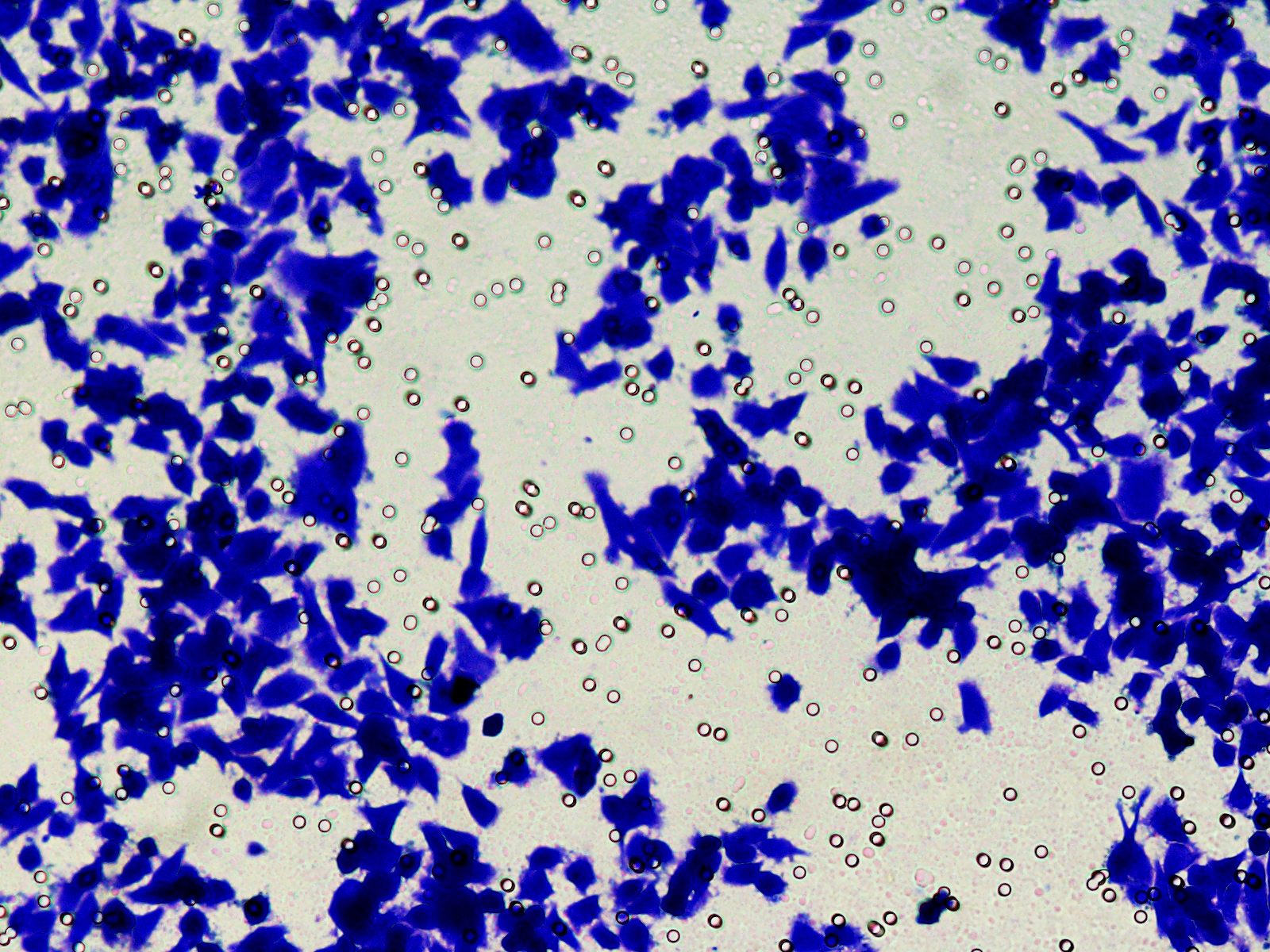

Supplement: S3 File — (ZIP) [file pone.0231923.s003.zip › Transwell/MKN45/Control/control-3.tif]

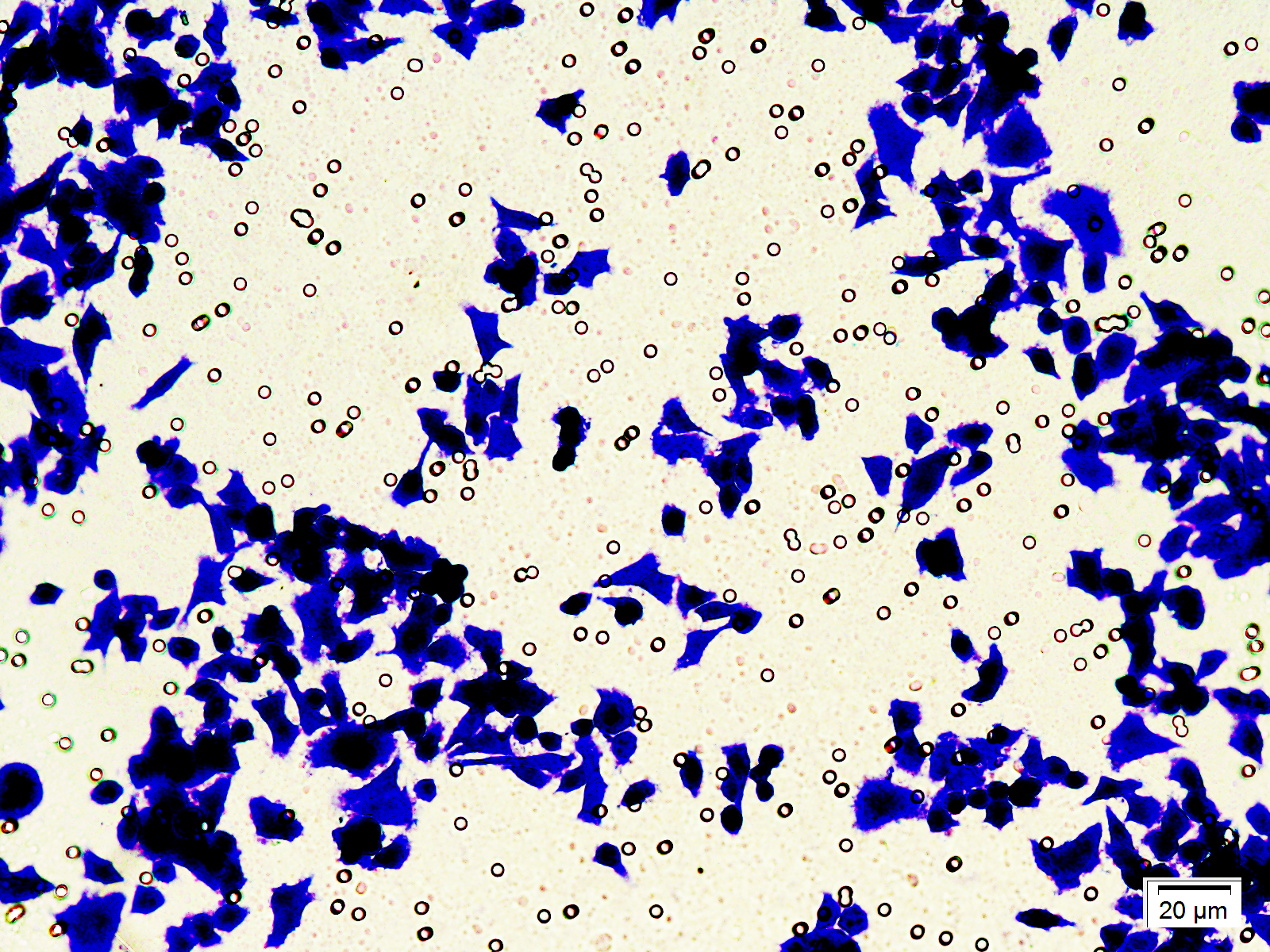

Supplement: S3 File — (ZIP) [file pone.0231923.s003.zip › Transwell/MKN45/TMTP1-DKK/TMTP1-DKK-1.tif]

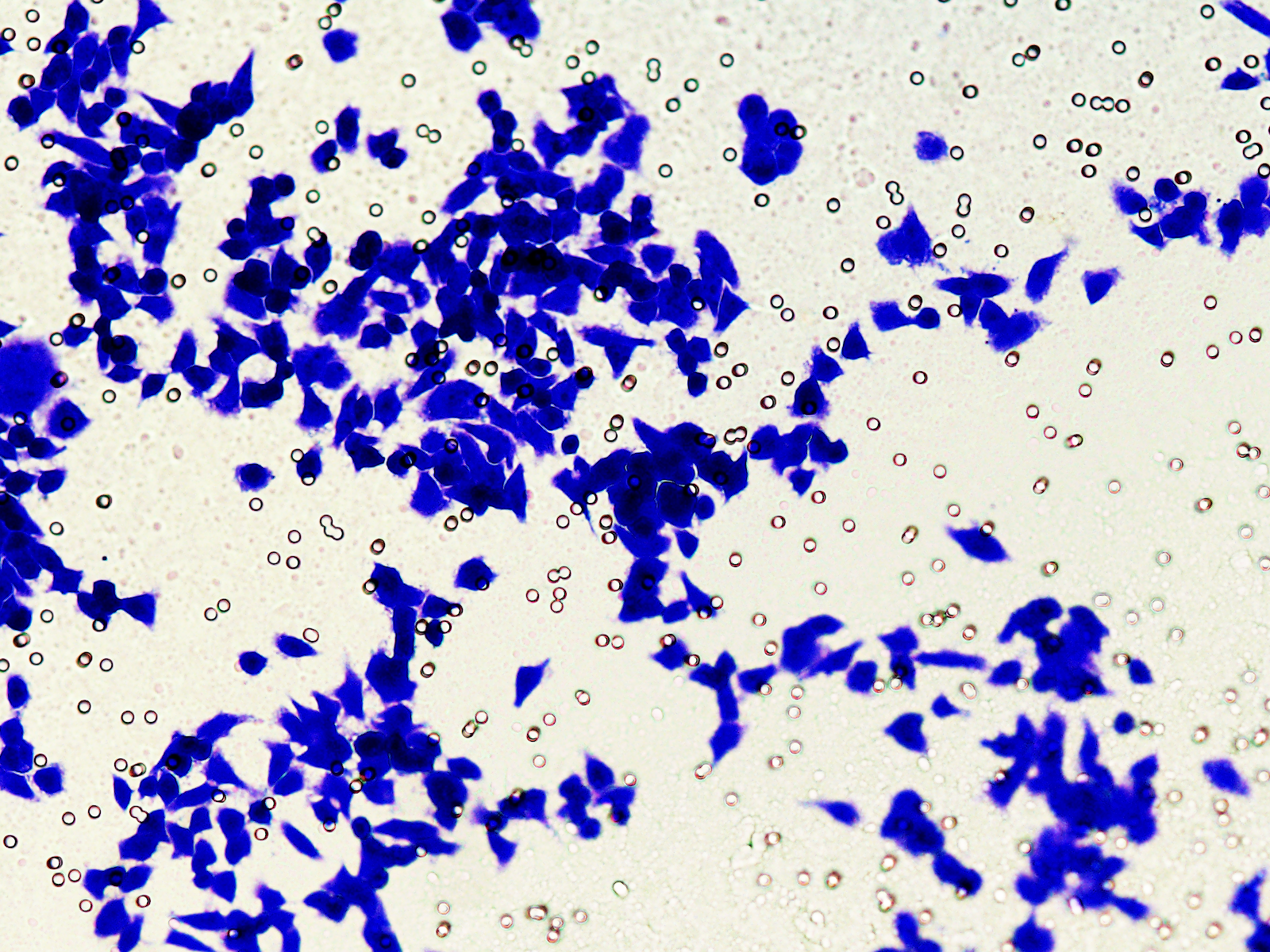

Supplement: S3 File — (ZIP) [file pone.0231923.s003.zip › Transwell/MKN45/TMTP1-DKK/TMTP1-DKK-2.tif]

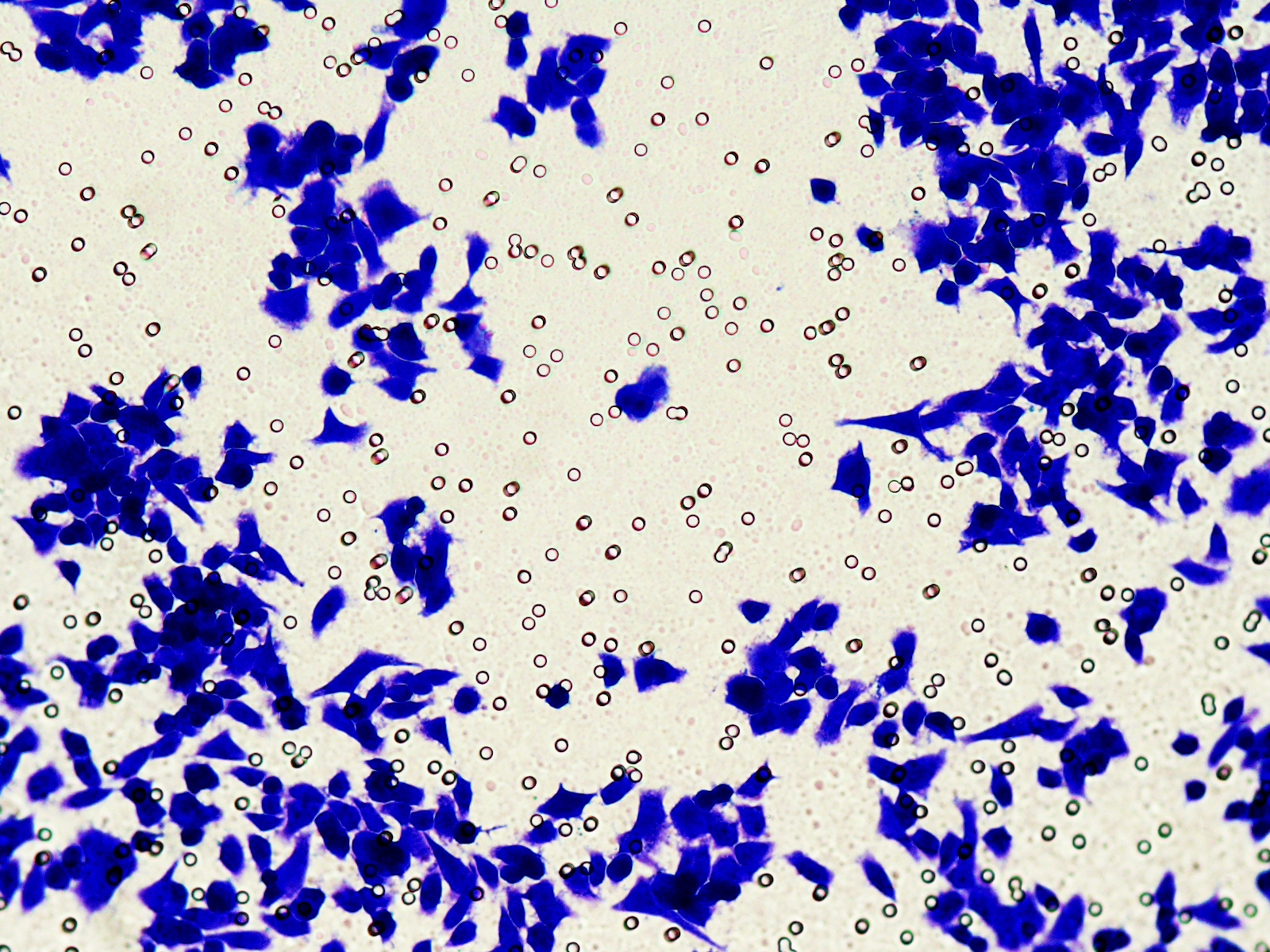

Supplement: S3 File — (ZIP) [file pone.0231923.s003.zip › Transwell/MKN45/TMTP1-DKK/TMTP1-DKK-3.tif]

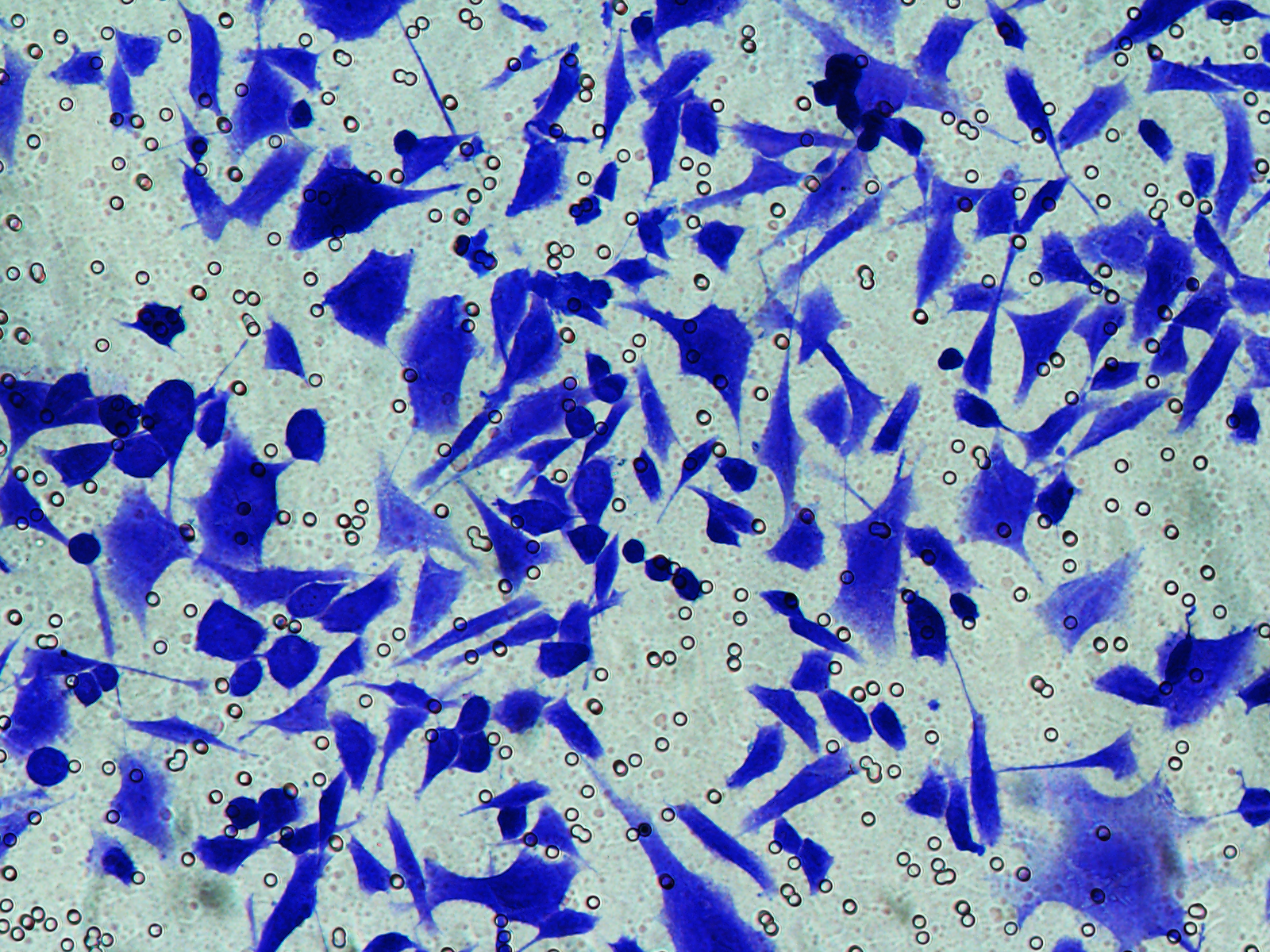

Supplement: S3 File — (ZIP) [file pone.0231923.s003.zip › Transwell/PC-3M-1E8/control/control-1.tif]

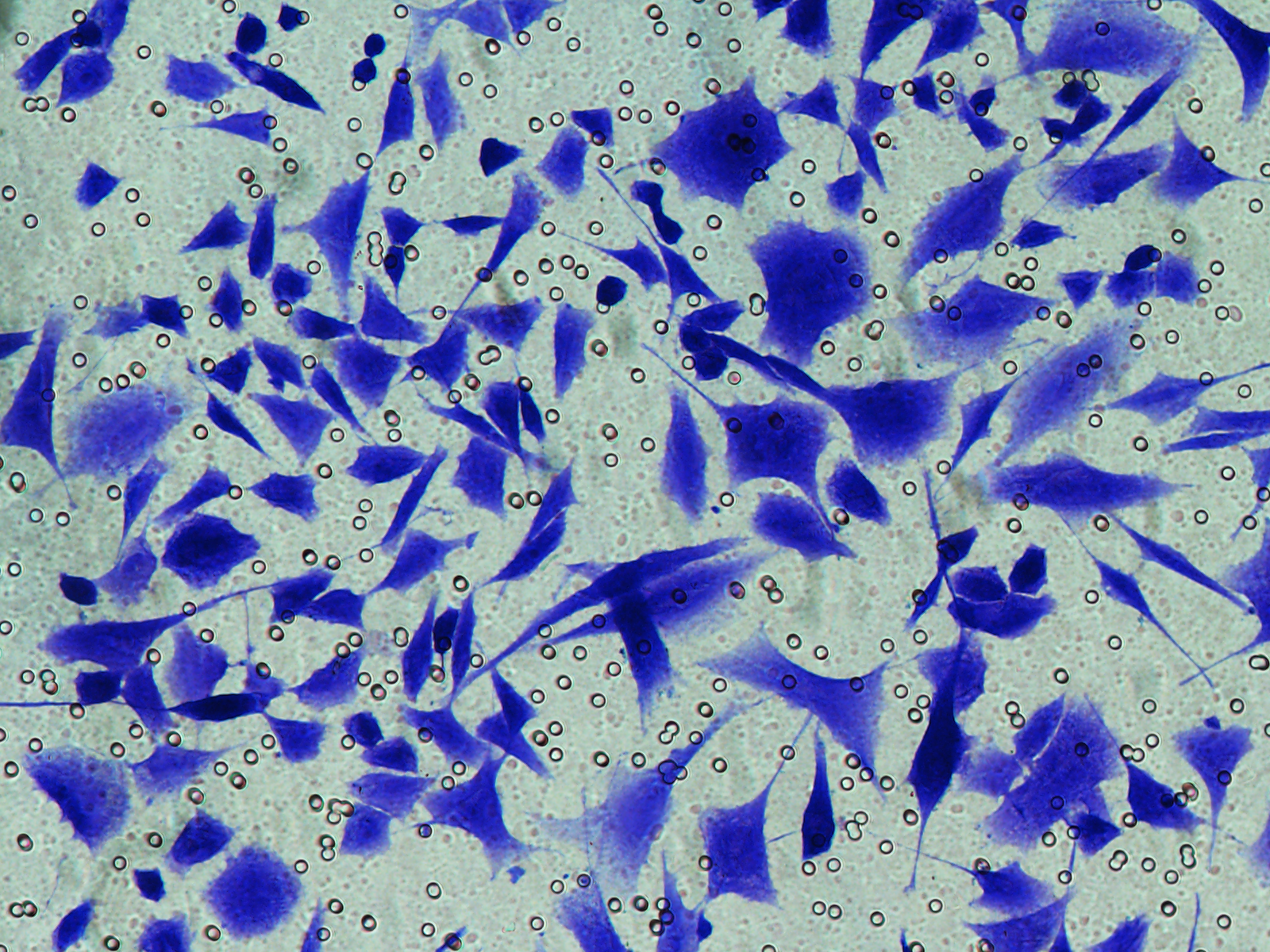

Supplement: S3 File — (ZIP) [file pone.0231923.s003.zip › Transwell/PC-3M-1E8/control/control-2.tif]

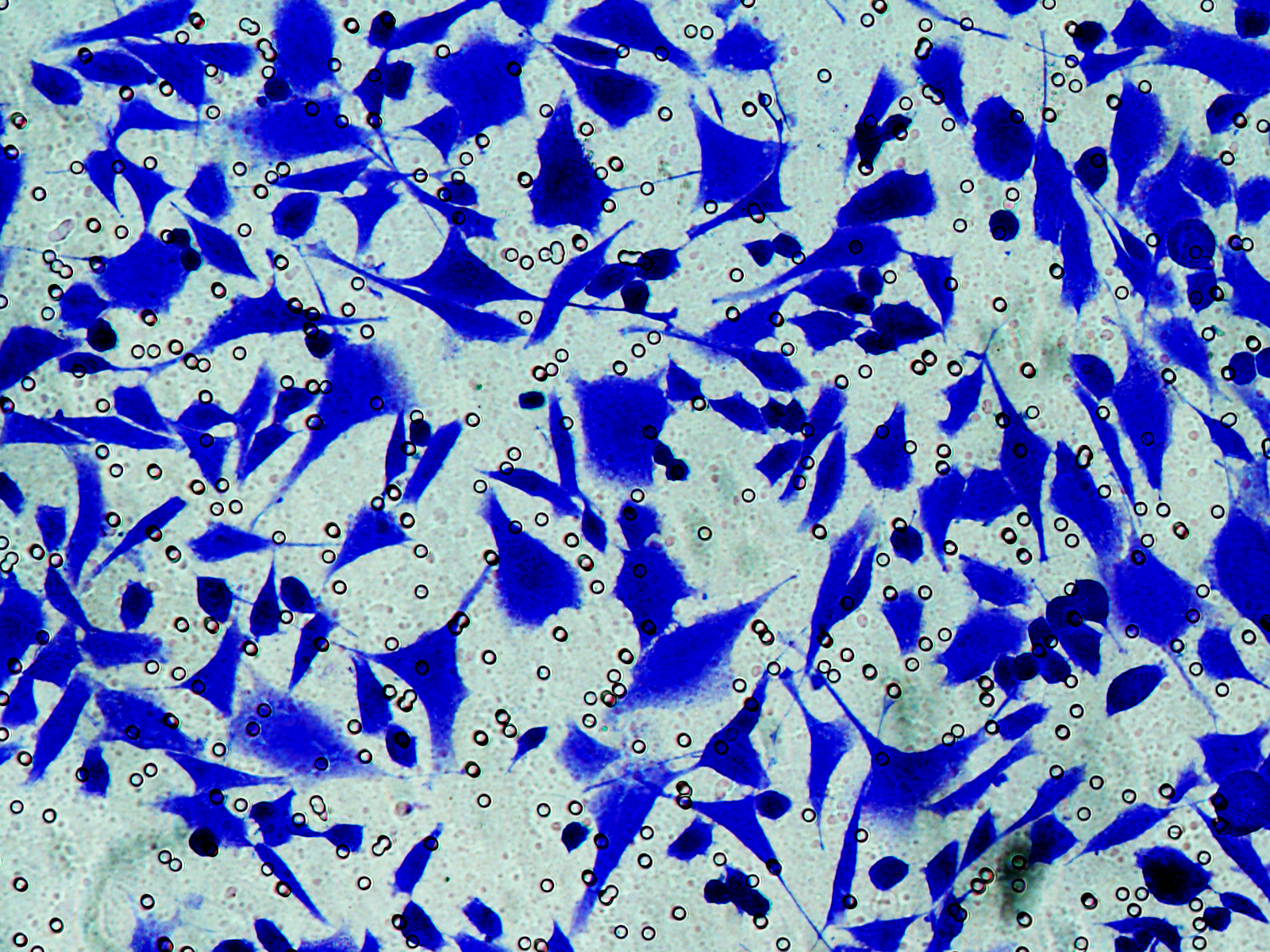

Supplement: S3 File — (ZIP) [file pone.0231923.s003.zip › Transwell/PC-3M-1E8/control/control-3.tif]

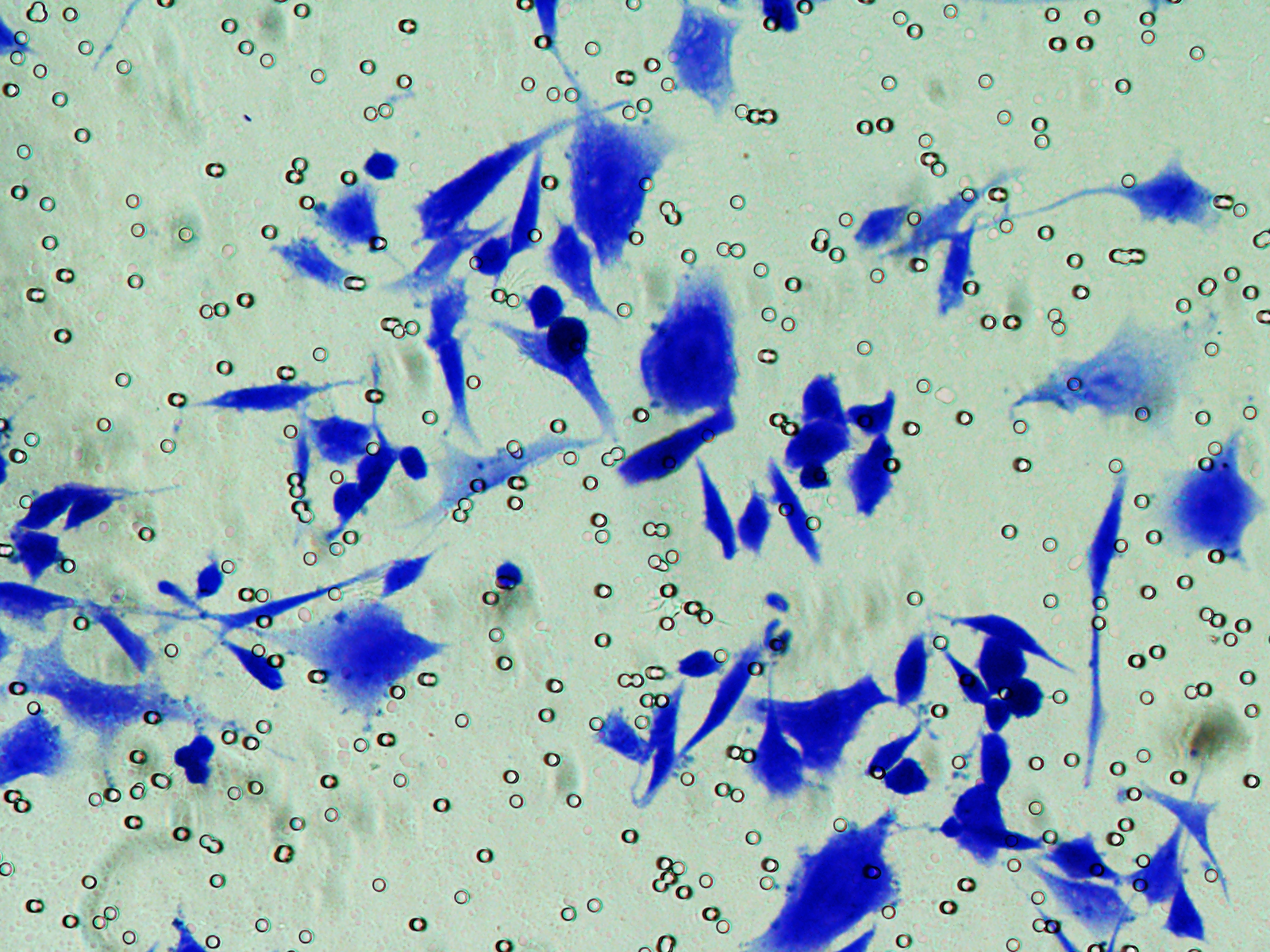

Supplement: S3 File — (ZIP) [file pone.0231923.s003.zip › Transwell/PC-3M-1E8/TMTP1-DKK/control-1.tif]

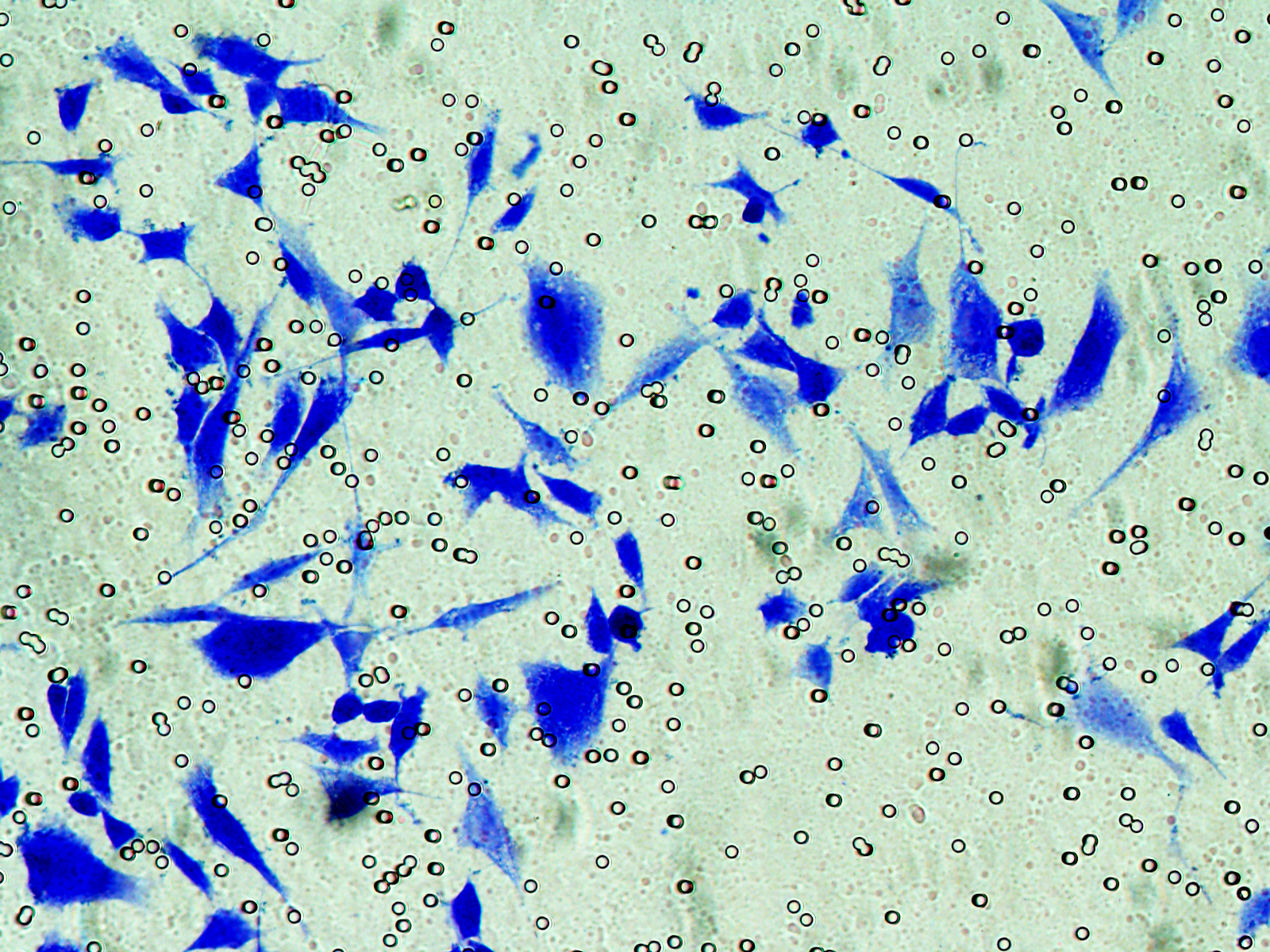

Supplement: S3 File — (ZIP) [file pone.0231923.s003.zip › Transwell/PC-3M-1E8/TMTP1-DKK/control-2.tif]

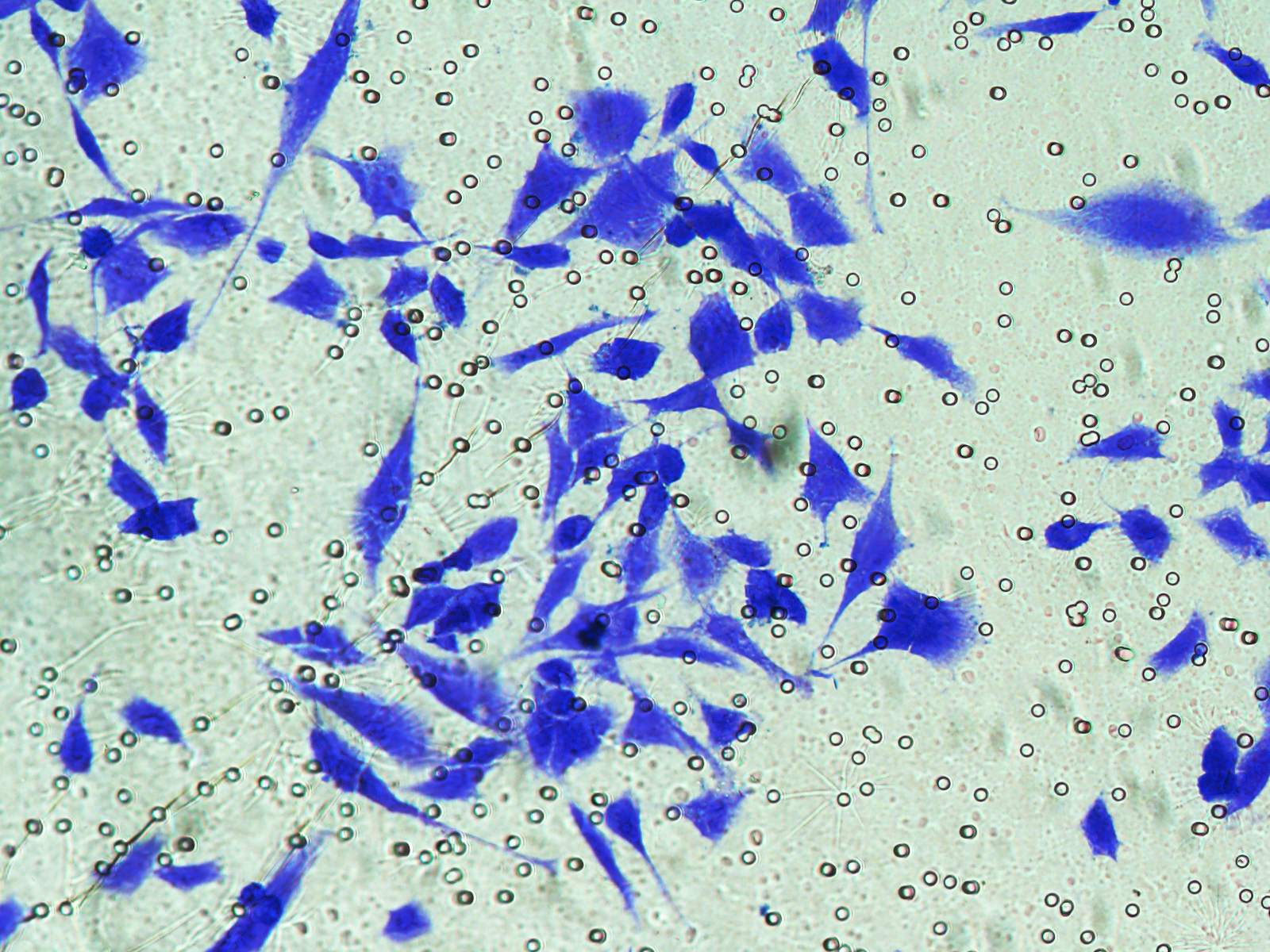

Supplement: S3 File — (ZIP) [file pone.0231923.s003.zip › Transwell/PC-3M-1E8/TMTP1-DKK/control-3.tif]
